# Unraveling the evolutionary dynamics of ancient and recent polypoidization events in *Avena* (Poaceae)

**DOI:** 10.1038/srep41944

**Published:** 2017-02-03

**Authors:** Qing Liu, Lei Lin, Xiangying Zhou, Paul M. Peterson, Jun Wen

**Affiliations:** 1 Key Laboratory of Plant Resources Conservation and Sustainable Utilization, South China Botanical Garden, Chinese Academy of Sciences, Guangzhou 510650, China; 2 University of Chinese Academy of Sciences, Beijing 100049, China; 3 Department of Botany, National Museum of Natural History, Smithsonian Institution, Washington, DC 20013-7012, USA

## Abstract

Understanding the diversification of polyploid crops in the circum-Mediterranean region is a challenging issue in evolutionary biology. Sequence data of three nuclear genes and three plastid DNA fragments from 109 accessions of *Avena* L. (Poaceae) and the outgroups were used for maximum likelihood and Bayesian analyses. The evolution of cultivated oat (*Avena sativa* L.) and its close relatives was inferred to have involved ancient allotetraploidy and subsequent recent allohexaploidy events. The crown ages of two infrageneric lineages (*Avena* sect. *Ventricosa* Baum ex Romero-Zarco and *Avena* sect. *Avena*) were estimated to be in the early to middle Miocene, and the *A. sativa* lineages were dated to the late Miocene to Pliocene. These periods coincided with the mild seasonal climatic contrasts and the Mediterranean climate established in the Mediterranean Basin. Our results suggest that polyploidy, lineage divergence, and complex reticulate evolution have occurred in *Avena*, exemplifying the long-term persistence of tetraploids and the multiple origins of hexaploids related to paleoclimatic oscillations during the Miocene-Pliocene interval in the circum-Mediterranean region. This newly-resolved infrageneric phylogenetic framework represents a major step forward in understanding the origin of the cultivated oat.

Genome duplication following hybridization (allopolyploidy) is common among flowering plants, and is found in nearly a quarter of Poaceae that provide crops and fuel worldwide^1^. Phylogenetic evidence from nuclear loci has accumulated to identify allopolyploidy events because they produce characteristic double-labelled phylograms in which allopolyploids appear more than once^2^. This approach does require sufficient depth of sequencing and the identification of paralogues produced by gene duplication events^3^.

The genus *Avena* L. (Poaceae) contains ca. 29 species exhibiting considerable morphological and ecological diversity in the Mediterranean Basin, Eastern Africa, Europe, Asia, and the Americas^4^,^5^. Based on glume shape, lemma apex, and the insertion of lemmatal awn, seven sections have been recognized for *Avena: Avenotrichon* (Holub) Baum, *Ventricosa* Baum ex Romero-Zarco, *Agraria* Baum, *Tenuicarpa* Baum, *Ethiopica* Baum, *Pachycarpa* Baum, and *Avena*^6^. The genus forms a polyploid series ranging from A- and C-genome diploids (2*x* = 14), AB- and A’C (DC)-genome tetraploids (4*x* = 28), to ACD-genome hexaploids (6*x* = 42)^7^. The A- and C-genome diploids are distinguished by the structural differentiation of isobrachial and heterobrachial chromosomes^8^, while the B and D genomes are not found in any extant diploids^9^,^10^.

Molecular data support a close relationship between D and A genomes^11^. Molecular and genome size analyses suggest that D-genome diploids hybridized with AC-genome tetraploids followed by chromosome doubling to form hexaploids^12^,^13^. Alternatively, the hexaploid D genome was inferred to originate from C-genome *A. clauda* Dur^14^ rather than from *A. canariensis* Baum & Raj. & Samp^8^. Recent molecular evidence suggest that three tetraploids *A. insularis* Ladiz., *A. maroccana* Grand., and *A. murphyi* Ladiz. may contain the D genome found in hexaploid oat^11^,^12^. A clear molecular delineation on D-genome origins would lead to a better understanding and utilization of genetic resources in *Avena*.

Cultivated oat offers a model for unraveling the dynamic evolutionary process of polyploid crops in the Mediterranean Basin^15^. Initial study of repeat sequences indicated that *A. strigosa* Schreb. DNA was homologous to the A-genome sequences of the cultivated oat^16^. Some studies proposed that *A. canariensis*^9^, *A. longiglumis* Dur^13^. or *A. weistii* Steud^17^. might be the A-genome progenitor. Recently, nuclear data demonstrated that the A genome evolved from multiple maternal lineages such as *A. damascena* Rajah & Baum, *A. hirtula* Lag., and *A. wiestii* Steud. rather than from one particular species^9^,^18^. Numerous intergenomic translocations complicate A-genome progenitor identification for the cultivated oat^8^,^12^,^16^. However, broader sampling of nuclear genes should make it possible to resolve this question.

Given chromosome structural differentiation, the C-genome origin of cultivated oat has been under intense scientific scrutiny. Eighteen chromosomes were involved in intergenomic translocations between C and A genomes of *A. sativa*^19^. Cytogenetic study indicated that none of the extant C-genome diploids could be the C-genome progenitor^20^. Plastid data supported that A’C(DC)-genome tetraploids served as the C-genome donors^21^,^22^, whereas nuclear data proposed that the C genome originated from a C-genome diploid (*A. clauda*)^18^. Thus, the C-genome ancestry of cultivated oat remains a challenging mystery.

The Mediterranean Basin, encompassing an area between 28°–48°N and 10°–39°E, is one of the 34 global biodiversity hotspots with c. 24,000 (10% of all seed) plant species^23^, and a diversification centre of *Avena* with 28 (96.55%) species (except for *A. abyssinica* Hochst.; Fig. 1)^5^,^24^,^25^. The origin of western Mediterranean dated to the Eocene (35 million years ago, mya), and the eastern Mediterranean was formed during the mid-Miocene (16 mya) by collision of Arabian and Eurasian tectonic plates, which led to the configuration of the modern Mediterranean Basin^26^. The mild seasonal contrasts were characterized by greater fluctuations in rainfall than in temperature during the early Miocene (23–16 mya), the repeated cooling events subsequently developed in the mid-Miocene (14–10 mya)^27^. Modern Mediterranean climate became established from 9–8 mya (onset of an arid climate) to 3.2–2.3 mya (onset of a seasonal climate)^28^. The mild climatic oscillation led to the extinction of tropical-subtropical floristic components (e.g., Taxodiaceae)^27^ together with the harsh climatic oscillation apparently contributing to the expansion of xerophytic taxa (e.g., *Anthemis*)^28^. The establishment of Mediterranean climate was considered to have triggered the speciation of C_3_ polyploid cool-season grasses, e.g., fodder ryegrasses^29^.

Here we sample the majority of *Avena* species (Supplementary Table S1^30^) and present a phylogenetic analysis with divergence time estimates based on nuclear and plastid sequences (Table S2^31^). The objectives are to elucidate infrageneric phylogenetic relationships within *Avena*, clarify A-, C-, and D-genome evolutionary history for the cultivated oat, and provide a hypothesis for the early diversification history of *Avena* in the circum-Mediterranean region.

## Results

### *PpcB1* sequence analysis

The aligned *ppcB1* matrix had 1017 characters, including exons 8 and 9, and intron 9; with the lengths of 783 bp, 54 bp, and 180 bp, respectively (Table S3). The *ppcB1* data provided 220 (21.63%) parsimony-informative characters. The maximum likelihood (ML) analyses and the Bayesian inference (BI) showed an identical topology for *Avena* (Fig. 2).

The monophyly of *Avena* received strong support (MLBP = 96%, PP = 1.00). Three clades and two nodes were observed in the *ppcB1* phylogram: A’C-PPI (*A. longiglumis*, A-type sequences of *A. agadiriana* and A’-type sequences of *A. maroccana* (PP = 0.98) (Supplementary Fig. S1); node A’C-PPII [*A. atlantica, A. damascena, A. longiglumis, A. wiestii*, and A’-type sequences of tetraploids (*A. agadiriana, A. insularis, A. maroccana*, and *A. murphyi*), and A- and A’-type sequences hexaploids (*A. fatua, A. hybrida, A. nuda, A. occidentalis, A. sativa*, and *A. sterilis*)] (Fig. S1); node AB-PPI [*A. brevis, A. canariensis, A. damascena, A. hirtula, A. hispanica, A. lusitanica, A. prostrata, A. strigosa, A. wiestii*, tetraploids (*A. abyssinica, A. barbata, A. vaviloviana* and *A. maroccana*) and hexaploids (without *A. sativa* and *A. occidentalis*)] (Fig. S2); A-PPI [*A. wiestii* and A’(D)-type sequences of hexaploids (without *A. fatua* and *A. nuda*)] (PP = 0.80) (Fig. S3); and A’C-PPIII [*A. hirtula*, C-genome diploids (*A. clauda, A. eriantha*, and *A. ventricosa*), and A’ and C-type sequences of tetraploids (*A. insularis, A. maroccana*, and *A. murphyi*) and hexaploids (without *A. nuda*)] (PP = 0.54) (Fig. S3). The clade A’C-PPI was sister to a single monophyletic lineage (PP = 0.62) containing nodes AB-PPI and A’C-PPII and clades A-PPI and A’C-PPIII in *Avena* (Fig. 2).

Three [A, A’(D), and C]-types of *ppcB1* sequences were identified for one accession of *A. sativa (Liu 273*), consistent with its hexaploid origin. These sequences fell into three distinct groups. In clade A’C-PPII, A’-type sequences of *A. sativa* clustered with tetraploids (*A. atlantica, A. agadiriana, A. insularis*, and *A. murphyi*) and hexaploids (without *A. nuda*) in subclade A’C-PPII-A1 (MLBS = 74%, PP = 0.54), whereas A-type sequence of *A. sativa* clustered with *A. longiglumis* in subclade A’C-PPII-A2 (MLBS = 94%, PP = 1.00) (Fig. S1). C-type sequences of hexaploids grouped with *A. hirtula*, C-genome diploids and three tetraploids (*A. insularis, A. maroccana*, and *A. murphyi*) in clade A’C-PPIII (Fig. S3). As for clade A-PPI, A’(D)-type sequence of *A. sativa* clustered with *A. wiestii* in subclade A-PPI-D (MLBS = 70%, PP = 0.94), which was labelled as “A’(D)” due to its distinct status in *Avena* (Fig. S3).

### *GBSSI* sequence analysis

The aligned *GBSSI* matrix had 1352 characters, including exons 9, 10, 11, 12, 13, and 14, and introns 8, 9, 10, 11, 12, 13, and 14, with the lengths of 53 bp, 81 bp, 194 bp, 88 bp, 129 bp, 22 bp, 47 bp, 148 bp, 153 bp, 127 bp, 154 bp, 152 bp, and 4 bp, respectively (Table S3). The *GBSSI* data provided 434 (32.1%) parsimony-informative characters. The ML and BI analyses generated different topologies for *Avena* (Figs 3 and 4).

The monophyly of *Avena* received strong support (MLBS = 94%, PP = 1.00) (Figs 3 and 4). In the ML analysis, three clades plus eight polyploids [A’(D)-type sequences of tetraploids *A. insularis* and *A. maroccana*, and hexaploids (without *A. nuda*)] were observed in the *GBSSI* tree: A’C-GBI [C-genome diploids, C-type sequences of tetraploids (*A. insularis, A. maroccana*, and *A. murphyi*) and hexaploids (without *A. nuda*)] (MLBS = 66%, PP = 1.00) (Fig. S4); AB-GBI [*A. atlantica, A. hirtula, A. longiglumis, A. wiestii*, A-type sequences of tetraploids (*A. abyssinica, A. barbata*, and *A. vaviloviana*) and hexaploid *A. fatua*] (MLBS = 100%, PP = 0.93) (Fig. S5); and AB&A’C-GBI [*A. atlantica, A. brevis, A. canariensis, A. damascena, A. hirtula, A. hispanica, A. longiglumis, A. lusitanica, A. strigosa, A. wiestii*, A-type sequences of tetraploids (*A. abyssinica, A. agadiriana, A. barbata*, and *A. vaviloviana*), A’-type sequences of *A. maroccana* and *A. murphyi* and hexaploids] (Fig. S6). The clade A’C-GBI was sister to a single lineage (PP = 0.98) containing clades AB-GBI and AB&A’C-GBI in *Avena* (Fig. 3).

In BI analyses, four clades plus eight polyploids [A’(D)-type sequences of tetraploids *A. insularis* and *A. maroccana*, and hexaploids (without *A. nuda*)] were observed in the *GBSS1* tree: A’C-GBI (C-type sequences of clade AC-GBI members in ML analysis) (MLBS = 66%; PP = 1.00) (Fig. S7); A’C-GBII [*A. brevis, A. canariensis, A. hirtula, A. hispanica, A. longiglumis, A. lusitanica, A. strigosa, A. wiestii*, A-type sequences of *A. agadiriana* and hexaploids (*A. hybrida, A. nuda*, and *A. sativa*), and A’-type sequences of *A. maroccana* and *A. murphyi*)] (PP = 0.50) (Fig. S8); AB-GBI [A-type sequences of clade AB-GBI members in ML analysis] (MLBS = 100%; PP = 0.94) (Fig. S9); and AB-GBII [*A. atlantica, A. damascena, A. hirtula, A. longiglumis, A. wiestii*, A-type sequences of three AB-genome tetraploids (*A. abyssinica, A. barbata*, and *A. vaviloviana*) and hexaploids (without *A. nuda* and *A. sterilis*)] (PP = 0.66) (Fig. S10). Clades A’C-GBII plus AB-GBII included the same members as clade AB&AC-GBI (without *A. sterilis*). Clade AB-GBI was sister to clade AB-GBII, and this group (PP = 0.66) plus clade A’C-GBII (PP = 0.50) was assigned to a single monophyletic lineage (PP = 0.98), which was sister to clade A’C-GBI. This large clade received strong support (MLBS = 94%, PP = 1.00) in *Avena* (Fig. 4).

Three [A, A’(D), and C]-types of *GBSSI* sequences were identified in four accessions of *A. sativa (Liu 272, 310, 311*, and *348*), consistent with its hexaploid origin. These sequences fell into three distinct groups. In clade A’C-GBI, C-type sequences of *A. sativa* clustered with C-genome diploids, C-type sequences of tetraploids (*A. insularis, A. maroccana*, and *A. murphyi*) and hexaploids (without *A. nuda*) (MLBS = 78%, PP = 1.00) (Figs S4 and S7). A-type sequences of *A. sativa* were inserted into clade AB&A’C-GBI and clade AB-GBII (Figs S6 and S10), respectively. However, A’(D)-type sequences of *A. sativa* were embedded within a single lineage containing clades AB-GBI and AB&A’C-GBI in the ML analysis (Fig. S6), and containing clades A’C-GBII, AB-GBI, and AB-GBII in BI analysis (Fig. S10).

### *Gpa1* sequence analysis

The aligned *gpa1* matrix had 1034 characters, including exons 10, 11, 12, introns 10, 11, and 12; with the lengths of 22 bp, 94 bp, 60 bp, 681 bp, 92 bp, and 85 bp, of which 137 (13.25%) were parsimony-informative. ML and BI analyses had an identical topology for *Avena* (Fig. 5).

The monophyly of *Avena* received strong support (MLBP = 100%, PP = 1.00). Seven clades were observed for the *gpa1* tree (Fig. S11): C-GPI (C-genome diploids) (MLBS = 100%, PP = 1.00); A’C-GPI [C-type sequences of tetraploids (*A. insularis, A. maroccana*, and *A. murphyi*) and five hexaploids (without *A. nuda*)] (MLBS = 88%, PP = 1.00); A’C-GPII [A-type sequences of *A. agadiriana*, and A’-type sequences of *A. insularis* and *A. murphyi*) and A’(D)-type sequences of four hexaploids (*A. fatua, A. occidentalis, A. sativa*, and *A. sterilis*)] (MLBS = 65%, PP = 0.96); A-GPI (*A. canariensis* and A-type sequence of *A. hybrida*) (MLBS = 96%, PP = 1.00); AB-GPI [diploids (*A. atlantica, A. damascena, A. hirtula*, and *A. wiestii*), A-type sequences of tetraploids (*A. abyssinica, A. barbata, A. vaviloviana*) and A’-type sequences of *A. maroccana*)] (MLBS = 73%, PP = 1.00); A’C-GPIII (*A. hirtula* and A’-type sequences of *A. maroccana*) (MLBS = 87%, PP = 1.00); and AB-GPII [*A. atlantica, A. brevis, A. damascena, A. hirtula, A. hispanica, A. longiglumis, A. lusitanica, A. strigosa*, and *A. wiestii*, A-type sequences of four AB-genome tetraploids and A’(D)-type sequences of *A. insularis* and hexaploids] (MLBS = 52%, PP = 0.93). Clades A-GPII, A-GPI, AB-GPI, A’C-GPIII, and A’C-GPII formed one monophyletic lineage (MLBS = 99%, PP = 1.00), and this lineage in turn was sister to clade A’C-GPI with strong support (MLBS = 92%, PP = 0.99), then the large group was sister to clade C-GPI with strong support (MLBS = 100%, PP = 1.00) (Fig. S11).

Two [A’(D)- and C-] types of *gpa1* sequences were identified in a single accession of *A. sativa (Liu 310*). These sequences fell into two distinct groups, with A’(D)-type sequence of *A. sativa* nested within clade AB-GPII, and C-type sequences of *A. sativa* nested within clade A’C-GPI (Fig. S11).

### Divergence times

The combined plastid data of 104 accessions comprised 2819 characters, of which 232 (8.23%) were parsimony-informative. The BEAST analysis generated a well-supported tree, which was identical to the topologies obtained from ML and BI analyses of *Avena* (Fig. 6). Two clades were recognized in the plastid phylogram: C-NRR (C-genome diploids *A. clauda, A. eriantha*, and *A. ventricosa*; MLBS = 99%, PP = 1.00); A’C-NRR [*A. brevis, A. canariensis*, A-type sequences of *A. barbata* and *A. agadiriana*, and A’(D)-type sequences of tetraploids (*A. insularis, A. maroccana*, and *A. murphyi*) and hexaploids] + AB-NRR [*A. atlantica, A. damascena, A. hirtula, A. longiglumis, A. lusitanica, A. prostrata, A. strigosa, A. wiestii*, A-type sequences of AB-genome tetraploids and A’-type sequence of *A. maroccana*) and hexaploids (without *A. sativa* and *A. occidentalis*)]. Clade A’C-NRR + AB-NRR (MLBS = 97%, PP = 0.96) was sister to clade C-NRR in *Avena* (Fig. 6). Here we discuss divergence times for the lineages of interest as shown in Table S4.

The uncorrelated-rate relaxed molecular clock suggests that the crown age of *Avena* was 20.04 [95% highest posterior density (HPD) 3.56–35.06] mya (node 1). This was also the stem ages for clades C-NRR and A’C-NRR + AB-NRR, whose crown ages were 10.71 (HPD: 1.62–20.25) and 14.54 (HPD: 2.68–25.02) mya, respectively (nodes 2 and 3). The crown age of clade A’C-NRR + AB-NRR was also the divergence time for nodes A’C-NRR and AB-NRR (nodes 4 and 8). The crown ages of the *A. sativa* lineages were 2.43, 2.46, and 2.97 mya (nodes 5, 6, and 7), respectively (Fig. 6).

## Discussion

### Infrageneric phylogeny and allopolyploidy events in *Avena*

Two strongly supported infrageneric lineages within *Avena* were identified by the plastid data: the C-genome diploid lineage (*Avena* sect. *Ventricosa*) containing *A. clauda, A. eriantha*, and *A. ventricosa* in clade C-NRR; and the A-genome diploid-polyploid lineage (*Avena* sect. *Avena*) containing other congeneric species in clade AB-NRR + A’C-NRR (Fig. 6). Members of C-genome diploid lineage were distributed from the south Mediterranean to the Irano-Turanian region^5^,^6^, and they were easily distinguished based on their unequal glumes^15^, fusiform caryopses with striate sculpturing^32^, and heterobrachial chromosomes with heterochromatin blocks along long-arm terminals^8^. Morphological, cytogenetic, and phylogenetic evidence supported recognizing this lineage as a distinct section, *Avena* sect. *Ventricosa*, which was embedded within clades A’C-PPIII (Fig. S3) and A’C-GBI based on nuclear data (Figs S4 and S7). In the *ppcB1* and *GBSSI* trees, *Avena* sect. *Ventricosa* shared a high degree of genetic similarity with C-type homoeologues of polyploids. Consequently, the ancestor of *Avena* sect. *Ventricosa* was probably the C genome donor for A’C(DC)-genome tetraploids and hexaploids.

*Avena* sect. *Avena* was proposed for the A-genome diploid-polyploid lineage including nodes with low support in the plastid tree (Fig. 6). Chromosome rearrangement had occurred since the divergence of *Avena* sect. *Avena* progenitors, leading to the divergence of A-genome constitution^4^,^10^,^30^, which could be divided into two groups in the section. The first group, As-genome diploids (*A. brevis, A. hispanica, A. strigosa, A. atlantica, A. hirtula, A. lusitanica*, and *A. wiestii*), *A. damascena* (Ad-genome), and *A. longiglumis* (Al-genome) clustered with three A’C(DC)-genome tetraploids (*A. insularis, A. maroccana*, and *A. murphyi*) in node A’C-PPII (Fig. S1); and the second group, As-genome diploids (*Avena brevis, A. hispanica, A. strigosa, A. atlantica, A. hirtula, A. lusitanica*, and *A. wiestii*), *A. canariensis* (Ac-genome), *A. damascena* (Ad-genome), and *A. prostrata* (Ap-genome) clustered with AB-genome tetraploids in node AB-PPI (Fig. S2), and As-genome diploids (*A. atlantica, A. hirtula*, and *A. wiestii*), *A. damascena* (Ad-genome), and *A. longiglumis* (Al-genome) clustered with AB-genome tetraploids in clade AB-GBII (Fig. S10). Phylogenetic relationships among the As-, Ad-, and Al-genome diploids and A’C-genome tetraploids, together with those of As-, Ac-, Ad-, Al-, and Ap-genome diploids and AB-genome tetraploids indicated that the close relatives of the A’C- and AB-genome tetraploids might be found within different A-genome groups based on *ppcB1* data. Therefore, we hypothesize that AB- and A’C(DC)-genome tetraploids originated from different A-genome diploid ancestors (Table S5^13^,^16^,^18^,^21^,^22^). Whole genome sequencing data including repetitive DNA might be able to detect the A (A’)-genome constitution in *Avena* tetraploids^12^.

Three secondary gene pool members, *A. insularis, A. maroccana*, and *A. murphyi* are native to the northwest Africa and adjacent environs (i.e., *A. insularis* in Sicily and Tunisia, *A. maroccana* in Moroccana, and *A. murphyi* in southern Spain and northern Morocco)^5^,^21^. They formed a clade A’C-GPI together with hexaploids in the *gpa1* tree (Fig. S11). In view of the chromosome pairing capacity between A’C(DC)-genome tetraploids and hexaploids^21^ and sequence-based diversity data^9^, the A’ and C genomes in the three tetraploids matched closest with D and C genomes in cultivated oat^10^. Since the As-, Ad-, and Al-genome diploids were involved in the A’C(DC)-genome tetraploid formation, it cannot be excluded that A’C(DC)-genome tetraploids originated from the ancient allotetraploidy events owing to the isolated phylogenetic positions of *A. maroccana* in clade A’C-PPI (Fig. S1), those of *A. insularis* inserted within a monophyletic lineage of the *GBSSI* tree (Fig. S8), and that of *A. murphyi* in clade A’C-GPI (Fig. S11). If this was the case, one would expect three or more ancient A-genome diploids to have participated in the origin of A’C(DC)-genome tetraploid. The three tetraploids have been reported as AC-genome-derived based on anonymous genotyping-by-sequencing (GBS) markers^9^, while the A’C(DC) designation of the tetraploids is fully compatible with our results together with another analysis based on GBS markers located on hexaploid chromosomes^10^.

Within the As-genome diploids, *Avena hispanica* was isolated from the closely related *A. hirtula* and *A. lusitanica* in the clade A’C-NRR + AB-NRR of plastid tree (Fig. 6). However, *A. lusitanica* (group 5) showed specific genetic divergence from *A. hirtula* and *A. hispanica* (group 3) in high-density GBS analysis^10^. Based on the length of lemma biaristulate tips (5–12 mm^6^) and the genome size (9.08 ± 0.11^12^), *A. hirtula* can be easily differentiated from the two As-genome diploids, that have a similar genome size to the smallest Ad-genome diploid *A. damascene*^12^. The incongruencies among morphological characters and genetic differences make the identification of the As-genome species challenging. *Avena lusitanica* and *A. hispanica* might represent ecotypes of *A. hirtula* found in the circum-Mediterranean, western Asia, and Europe^5^,^33^.

### Allohexaploid origin of *Avena sativa*

Two distinct steps were inferred for the formation of the cultivated oat. The first step includes the ancient allotetraploidy events involving the hybridization between the ancient A’(or diverged A)- and C-genome diploid ancestors to form A’C (now called DC)-genome tetraploids. The second step includes subsequent recent allohexaploidy events involving hybridization between DC-genome tetraploids and the more recent A-genome diploid progenitors to form the extant ACD-genome hexaploids^18^. The close relationship between the genetically homogeneous *Avena* sect. *Ventricosa* and the C-copy sequences of A’C-genome tetraploids plus hexaploids was a novel discovery which suggested their C-genome donor to be the ancestor of *Avena* sect. *Ventricosa*. This was consistent with the hypothesis that the paleotetraploidy events pre-dated and potentially triggered divergence of the extant A’C(DC)-genome tetraploids in narrow ranges of the Mediterranean Basin^9^. In the *gpa1* tree, A’C(DC)-genome tetraploids together with hexaploids comprised the clade A’C-GPI (Fig. S11). Therefore, the nuclear data provided robust evidence for the designated D and C genomes in cultivated oat, matching closest with A’(D)- and C-genome in *A. insularis, A. maroccana*, and *A. murphyi*, and the A-genome designation matches better with the extant A-genome diploids in *Avena*.

The close relationships among three A-genome diploids and cultivated oat were observed in the *ppcB1* tree, i.e., *A. atlantica, A. longiglumis*, and *A. wiestii* were embedded within the A’C-PPII-A1, A’C-PPII-A2, and A-PPI subclades (Figs S1 and S3). The IGS-RFLP dendrogram suggested that *A. atlantica* should be placed within the cluster containing polyploids rather than within the *A. strigosa* cluster^13^, showing that *A. atlantica* has genetic dissimilarities with *A. strigosa*^34^. *Avena longiglumis* formed a strongly supported subclade A’C-PPII-A2 with *A. sativa* (Fig. S1). In addition, two new *ppcB1* clones of *A. wiestii (Rawi 11581*, US!) located in node A’C-PPII and clade A-PPI (Figs S1 and S3), together with two reported *FL intron2* clones (*CIav 9053*)^18^ indicate that the coexistance of diploid and tetraploid forms for *A. wiestii* is certainly different from other As-genome diploids. Although the different genome forms of *A. wiestii* were close in genome size^12^, the intraspecific differences between *A wiestii* deserves further investigation. Two plausible explanations can be proposed for the ploidy level of allelic variation found in *A. wiestii*. First, the three diploids may have arisen by allopolyploidy and subsequent unequal diploidization led to heterozygotes. Second, introgression may have brought about very subtle morphological and genetic changes in *Avena* (Fig. 2), because stabilized introgressant species were more common than cases of dispersed introgression involving extensive gene flow among distinct species^35^. The two explanations are not mutually exclusive, such as *Leucaena*^36^ sharing unequal diploidization and introgression processes. The paternally inherited genome of an allopolyploid is usually more prone to genetic change than the maternally derived genome according to the nuclear cytoplasmic interaction hypothesis^37^. In support of this hypothesis, it has been proposed that *A. atlantica, A. longiglumis*, or *A. wiestii* might carry the diverged A-genomes because considerable allelic variation was detected in the *ppcB1* and *FL int2* data^18^.

Based on *ppcB1* and cytogenetic data, a close phylogenetic relationships between the A and D genomes substantiates the multiple origins for cultivated oat^19^. However, the integrated theory for the long-term evolutionary impact of recurrent polyploidy was unclear for hexaploid divergence in *Avena*. Based on the level of genetic variation, it is logical to postulate that recurrent polyploidy from genetically distinct diploid progenitors would introduce genetic variation into hexaploids. Nuclear data have demonstrated that recurrent polyploidy can lead to hexaploids being reproductively isolated to varying degrees. Six hexaploids were found within A’C-PPIII, AB&A’C-GBI, AB-GPII clades, while some hexaploids were dispersed within other clades in the nuclear gene trees. For example, *Avena nuda* is morphologically distinct with falling caryopses, but it was independently inserted within node AB-PPI and clades AB-GBII and A’C-GBII, demonstrating varying degrees of interfertility when compared with *A. sativa*^15^. Clearly six hexaploids cannot be regarded as a single species designated as *A. sativa*^38^, especially for wild hexaploids—*A. fatua, A. sterilis, A. hybrida*, and *A. occidentalis*, each adapted to respective microenvironments in the circum-Mediterranean region^33^. *Avena* provided a great model for studying polyploidy, especially concerning the evolutionary and genetic processes associated with extensive intergenomic translocations^30^ and northward diffusion into cooler areas^33^ over a time scale of c. 20 mya (Fig. 6). Future studies of *Avena* need to investigate the unique and conserved genomic signatures using phylogenomics^39^,^40^.

### Paleoclimatic hypothesis for the lineage divergence of *Avena*

It has been proposed that the Miocene-Pliocene interval was a key period in the origin of Mediterranean temperate plants and involved two major climatic oscillations^41^. The former comprised mild seasonal climatic contrasts that resulted from rainfall decreasing and repeated cooling events during the early to middle Miocene; and the latter was characterized by a high seasonal Mediterranean climate resulting from the onset of aridity and seasonality during the late Miocene to Pliocene^27^. During these mild climatic contrasts, shifts in vegetation from subtropical forest to annual grasslands occurred in the Mediterranean Basin^29^. The resultant habitat heterogeneity may have had lasting impact on the genetic and phenotypic divergence of major lineages in *Avena*^27^. Major lineages in *Avena* are distinguished by ecological differentiation: *Avena* sect. *Ventricosa* is distributed in calcareous rocky plateaus or mountain grassland habitats; and *Avena* sect. *Avena* is distributed in carbonate sands or semi-desert habitats in the circum-Mediterranean region. The crown ages of these two lineages are estimated at 14.54 (HPD: 2.68–25.02) and 10.71 (HPD: 1.62–20.25) mya, respectively (Fig. 6). These periods coincide with mild seasonal climatic contrasts that occurred during the early to middle Miocene. It appears a temporal relationship exists between the mild seasonal climatic contrasts and the divergence of major lineages in *Avena*.

Cultivated oat may have arisen multiple times in response to selection pressure such as geographic isolation. The long-term aridity of the Mediterranean Basin summer became more severe along a south-eastern to north-western gradient during the late Miocene to Pliocene^27^, leading to the domination of open habitats by C_3_-pooid grasses^42^. The increased colonization capacity of cultivated oat may be strongly linked to hybridization between diploid and tetraploid progenitors followed by chromosome duplication. Recurrent polyploidization events in the *Avena sativa* lineages (nodes 5, 6, and 7) seem to correlate with highly seasonal climatic oscillation. Geographic isolation might have contributed to genetic differentiation in the progenitor-derivative species pair, the presumed D(or A’)-genome progenitors having disjunct distributions in the Mediterranean region (e.g., *A. atlantica* was endemic to Morocco, *A. wiestii* was endemic to the east Mediterranean, east Saharo-Arabian, and Irano-Turanian, and *A. longiglumis* was endemic to the west-south-east Mediterranean and Saharo-Arabian)^5^. The once extensive distribution of the narrow-endemic A’C(DC)-genome tetraploids underwent contraction. Hybridization might have been a key element in the successful spread of cosmopolitan cultivated oat as a result of incorporation of locally adapted genes from different progenitor genomes. If this was the case, then the initial hybridization must have pre-dated the formation of modern Mediterranean region^26^, which isolated *A. wiestii* (eastern-most) from *A. atlantica* (western-most). Therefore, the independent hexaploidy events of cultivated oat were modulated by harsh climatic oscillation, thus *A. sativa* was able to adapt to new habitats.

*Avena* represents a remarkable model to study because its history of polyploidy, lineage divergence, and complex reticulate evolution. The complex evolution of cultivated oat and its close relatives involved paleotetraploidy events between the ancient A(or A’)- and C-genome diploid ancestors and subsequent recent allohexaploidy events between A’C(DC)-genome tetraploids and the more recent A-genome diploid progenitors. The pattern of recurrent polyploidizations in *Avena* and their temporal relationships with paleoclimatic oscillations is unparalleled among polyploid crops occurring in the circum-Mediterranean region^4^,^43^.

## Methods

### Taxon sampling and data collection

Eighty-nine accessions of 27 species were sampled to represent the morphological diversity and geographic range of six sections in *Avena*^5^, together with outgroups comprising 20 accessions of 16 species from seven allied genera (Supplementary Table S1^30^) based on the recent phylogeny and classification of Poaceae^44^. Leaf material was obtained from seedlings and herbarium specimens.

Three low-copy nuclear genes, *phosphoenolpyruvate carboxylase B1 (ppcB1*), *granule-bound starch synthase I (GBSSI*) and *G protein alpha subunit 1 (gpa1*), were used. The *ppcB1* gene encodes *PEPC* enzyme for the oxaloacetate replenishment of the tricarboxylic acid cycle in C_3_ plants^45^, the *GBSSI* gene encodes *GBSSI* enzyme for the amylose synthesis in plants^46^, and the *gpa1* gene encodes a G-protein α subunit for signal transduction in flowering plants^47^. These loci have previously been used for accurate phylogenetic assessments in Poaceae^2^,^47^,^48^. Based on genome-wide studies on cereal crops, the three loci appear to be on different chromosomes^4^,^48^,^49^, thus each of nuclear markers can provide an independent phylogenetic estimate.

Genomic DNA was extracted following Liu *et al*^31^. and 864 new sequences were generated for nuclear (*ppcB1, GBSSI*, and *gpa1*) and plastid (*ndhA* intron, *rpl32-trnL*, and *rps16* intron) fragments, which were amplified using designed or published primers and protocols listed in Table S2^31^. Amplified products were purified using polyethylene glycol (PEG) precipitation protocols and sequenced using an ABI PRISM 3730XL DNA Analyzer (Applied Biosystems, Foster City, CA, USA). For accessions that unsuccessfully underwent direct sequencing, the purified PCR products were cloned into pCR4-TOPO vectors and transformed into *Escherichia coli* TOP10 competent cells following the protocol of TOPO TA Cloning Kit (Invitrogen, Carlsbad, CA, USA). The resulting sequences were edited using Sequencher v.5.2.3 (Gene Codes Corp., Ann Arbor, MI, USA) and aligned with MUSCLE v.3.8.31^50^, followed by manual adjustment in SE-AL v.2.0a11 (http://tree.bio.ed.ac.uk/software/seal/). All sequences were deposited in GenBank (KT452936–453223, KT723464–724040).

### Phylogenetic analyses

Phylogenetic analyses were performed using maximum likelihood^51^ and Bayesian inference^52^. Nucleotide substitution models were selected based on the Akaike Information Criterion determined by Modeltest v.3.7^53^. ML and bootstrap analyses (MLBS) were performed using the best-fit model (Table S3) for 1,000 bootstrap replicates in GARLI v.0.96^54^, with runs set for unlimited generations, and automatic termination following 10,000 generations without significant topological change (lnL increase of 0.01). The output file containing the best trees for bootstrap reweighted data was then read into PAUP* v.4.0b10^55^ where the majority-rule consensus tree was constructed to calculate MLBS.

**Figure 1:**
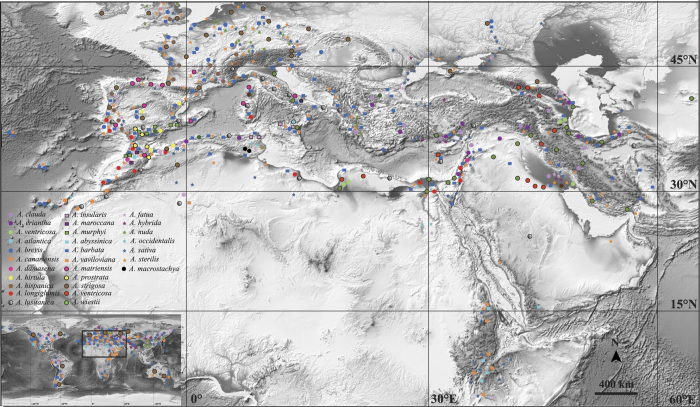
The diversification centre for 28 species of *Avena* (only *A. abyssinica* in eastern Africa and western Asia)^5^,^24^,^25^ in the Mediterranean Basin using software Adobe Photoshop CS6. The background map was downloaded from http://www.ngdc.noaa.gov/mgg/global/global.html (Amante C, Eakins BW. ETOPO1 1 Arc-Minute Global Relief Model: Procedures, Data Sources and Analysis. NOAA Technical Memorandum NESDIS NGDC-24. National Geophysical Data Center, NOAA. Doi:10.7289/V5C8276M, March 2009).

**Figure 2:**
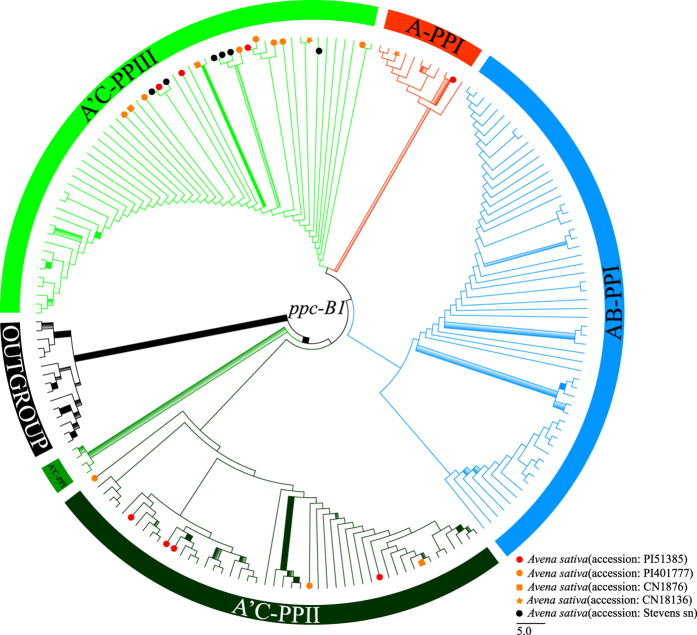
Maximum likelihood tree of *Avena* inferred from nuclear *ppcB1* data including three clades (A-PPI in red, A’C-PPI in green, and A’C-PPIII in light green) and two nodes (AB-PPI in blue and A’C-PPII in dark green). Branch thickness indicate maximum likelihood bootstrap support/Bayesian posterior probability (MLBS/PP): thickest solid = MLBS ≥ 90% and PP ≥ 0.90; thickest shadow = MLBS ≥ 90% or PP ≥ 0.90; thick solid = 89% ≥ MLBS ≥ 70% and 0.89 ≥ PP ≥ 0.70; thick shadow = 89% ≥ MLBS ≥ 70% or 0.89 ≥ PP ≥ 0.70; and thin solid = 69% ≥ MLBS ≥ 50% and 0.69 ≥ PP ≥ 0.50). Red, orange, and black of terminal symbols (circle, square, and star for different accessions) represent thrice, twice, and once clade/node appearance of the cultivated oat. Terminal taxon names and branch support values are shown in Figs S1–S3.

**Figure 3:**
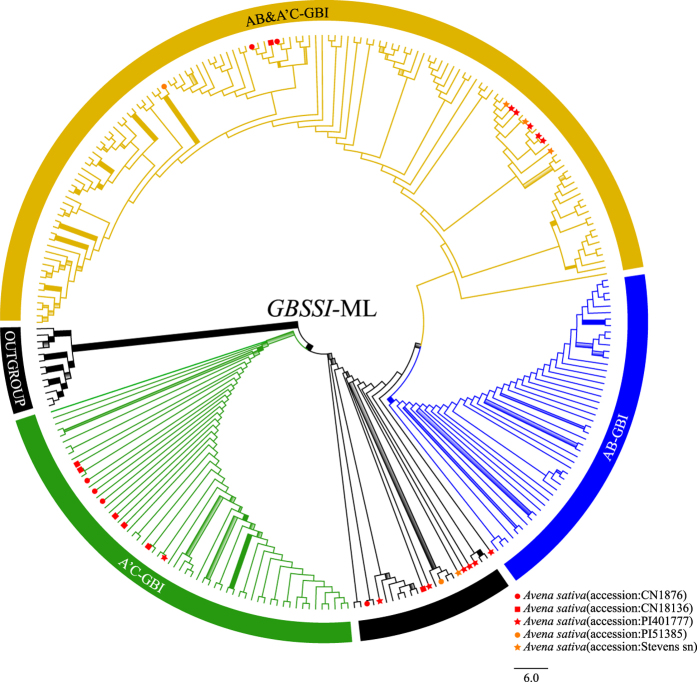
Maximum likelihood tree of *Avena* inferred from nuclear *GBSSI* data including three clades (AB-GBI in blue, A’C-GBI in green, and AB&A’C-GBI in brown) plus eight polyploids in unmarked black. Explanation of branch thickness and colorful terminal symbols refer to Fig. 2. Terminal taxon names and branch support values are shown in Figs S4–S6.

**Figure 4:**
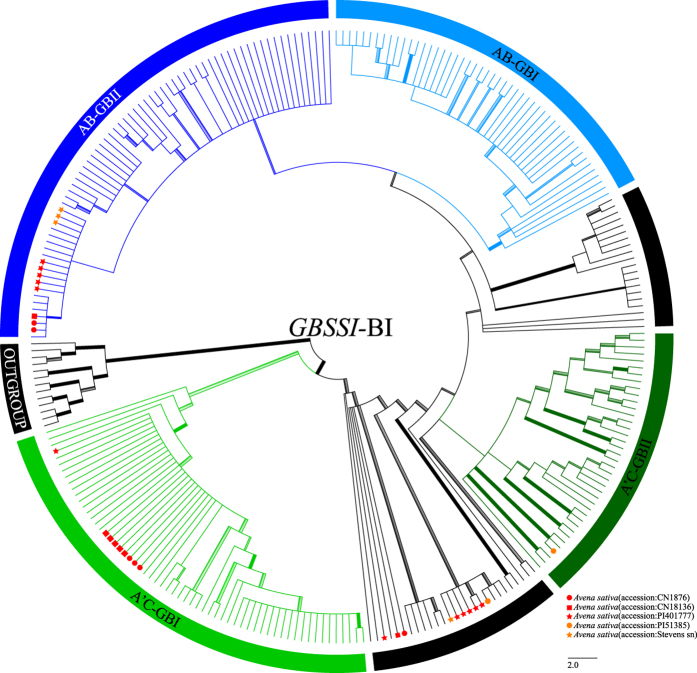
Bayesian inference phylogram of *Avena* inferred from nuclear *GBSSI* data including four clades (AB-GBI in light blue, AB-GBII in blue, A’C-GBI in green, and A’C-GBII in green) plus eight polyploids in unmarked black. Explanation of branch thickness and colorful terminal symbols refer to Fig. 2. Taxon names and branch support value are shown in Figs S7–S10.

**Figure 5:**
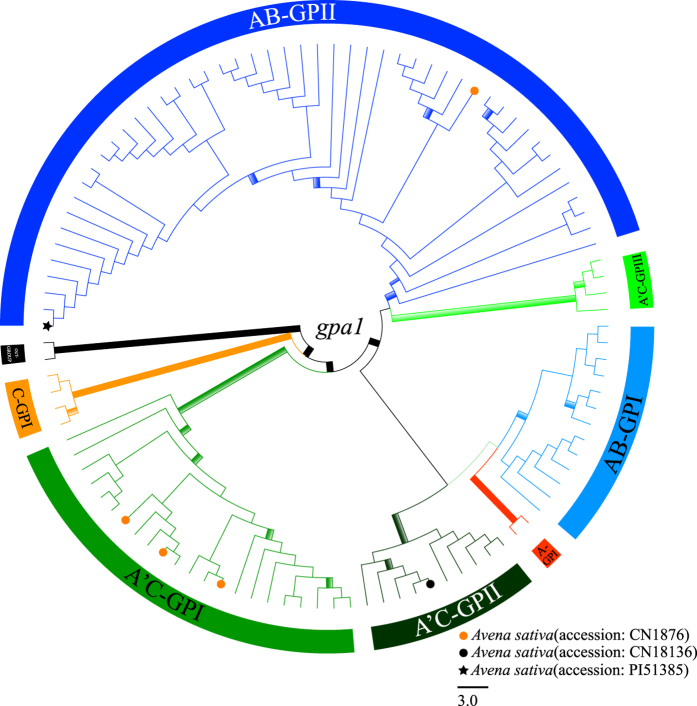
Maximum likelihood tree of *Avena* inferred from nuclear *gpa1* data including seven clades (A-GPI in red, C-GPI in brown, AB-GPI in light blue, AB-GPII in blue, A’C-GPI in green, A’C-GPII in dark green, and A’C-GPIII in light green). Explanation of branch thickness and colorful terminal symbols refer to Fig. 2. Taxon names and branch support value are shown in Fig. S11.

**Figure 6:**
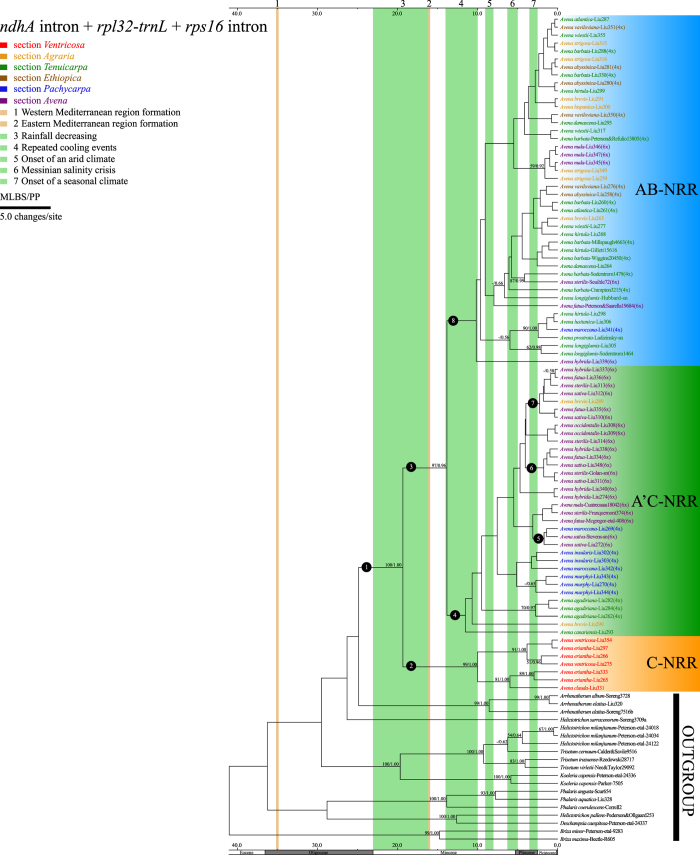
Chronogram of *Avena* and its close relatives based on plastid (*ndhA* intron, *rpl32-trnL*, and *rps16* intron) data including two clades (C-NRR and A’C-NRR + AB-NRR) inferred from BEAST. Numbers above the branches are MLBS/PP. Taxon labels are in the format: *Avena vaviloviana*-Liu351 (4x), where *Avena vaviloviana* indicates that the sequence belongs to the species; Liu351 indicates voucher; (4x) indicates that the species is tetraploid. Coloured taxon labels correspond to sections. Node number indicates the lineages of interest (Table S4).

BI analyses were conducted in MrBayes v.3.2.1^56^ using the best-fit model for each nuclear and the combined plastid loci (Table S3). The Bayesian Markov Chain Monte Carlo (MCMC) algorithm was run for 30 million generations with four incremental chains starting from random trees and sampling one out of every 1,000 generations. Convergence between runs and the choice of an appropriate burn-in value were assessed by comparing the traces using Tracer v.1.5 (http://tree.bio.ed.ac.uk/software/tracer). All resulting trees were then combined with LogCombiner v.1.6.1 (http://beast.bio.ed.ac.uk/) with 25% burn-ins. The remaining trees (c. 45,000) were used to calculate the Bayesian posterior probabilities (PP) for internal nodes. Data sets and phylogenetic trees are available at TreeBase (http://treebase.org, study no. TB2: S18544) (Reviewer access URL: http://purl.org/phylo/treebase/phylows/study/TB2:S18544. Figures 1, 2, 3, 4, 5, 6 (Supplementary Figs S1–S11) were prepared using Photoshop CS6 v.13.0 (Adobe, San Jose, CA, USA).

### Divergence time estimation

The molecular dating analyses employed plastid markers a strict molecular clock model was rejected at a significance level of 0.01 (LR = 963.1856, d.f. = 102, *P* < 0.01) based on a likelihood ratio test^51^. A Bayesian relaxed clock model was implemented in BEAST v.1.8.2^56^ to estimate divergence times in *Avena*. Three plastid markers were partitioned using BEAUTI v.1.8.2 (within BEAST) with the best fit model determined by Modeltest v.3.7 (Table S3). The stipoid-Pooideae lineage including *Avena* plus outgroups was dated to be 49.71 mya based on eight phytolith fossils, and thus the crown age of *Avena* plus outgroups was set at 49.71 mya since fossil surveys provide no evidence of an earlier date for the origin of the stipoid-Pooideae lineage during the late Eocene^57^.

A Yule tree prior, linked uncorrelated lognormal relaxed clock model, and default operators were defined in the BEAST xml input file. After optimal operator adjustment as suggested by the output diagnostics from preliminary BEAST runs, two independent MCMC runs were performed for 30 million generations, each run sampling every 1,000 generations with 25% burn-ins. All parameters had a potential scale reduction factor that was close to one, indicating that the posterior distribution had been adequately sampled. A 50% majority rule consensus from the retained posterior trees (c. 45,000) of three runs was obtained using TreeAnnotator v.1.8.2 (within BEAST) with a PP limit of 0.5 and mean lineage heights. The convergence between two runs was checked using the “cumulative” and “compare” functions in AWTY^58^.

## Additional Information

**How to cite this article**: Liu, Q. *et al*. Unraveling the evolutionary dynamics of ancient and recent polypoidization events in Avena (Poaceae). *Sci. Rep.* **7**, 41944; doi: 10.1038/srep41944 (2017).

**Publisher's note:** Springer Nature remains neutral with regard to jurisdictional claims in published maps and institutional affiliations.

## Supplementary Material

Supplementary Information

Supplementary Figs. S1-S11
