# Supplementary material for: Unraveling the evolutionary dynamics of ancient and recent polypoidization events in Avena (Poaceae)
Source: Sci Rep. 2017 Feb 3;7:41944. doi: 10.1038/srep41944 (PMC5291219; doi:10.1038/srep41944)
Supplement: Supplementary Information [file srep41944-s1.doc]

**Supplementary Information**

**Unraveling the evolutionary dynamics of ancient and recent polyoidization events in *Avena* (Poaceae)**

Qing Liu^1^, Lei Lin^1,2^, Xiangying Zhou^1,2^, Paul M. Peterson^3^, and Jun Wen^3^

^1^Key Laboratory of Plant Resources Conservation and Sustainable Utilization, South China Botanical Garden, Chinese Academy of Sciences, Guangzhou 510650, China;

^2^University of Chinese Academy of Sciences, Beijing 100049, China;

^3^Department of Botany, National Museum of Natural History, Smithsonian Institution, Washington, DC 20013-7012, USA.

Correspondence and requests for materials should be addressed to Q.L. ([liuqing@scib.ac.cn](mailto:liuqing@scib.ac.cn)) or J.W. ([wenj@si.edu](mailto:wenj@si.edu))

Table of Contents

**Supplemental Figure S1.** Maximum likelihood phylogeny of clade A’C-PPI and node A’C-PPII of *Avena* inferred from nuclear *ppcB1* data (Figure 2).

**Supplemental Figure S2.** Maximum likelihood phylogeny of node AB-PPI of *Avena* inferred from nuclear *ppcB1* data (Figure 2).

**Supplemental Figure S3.** Maximum likelihood phylogeny of clade A’C-PPIII of *Avena* inferred from nuclear *ppcB1* data (Figure 2).

**Supplemental Figure S4.** Maximum likelihood phylogeny of clade A’C-GBI of *Avena* inferred from nuclear *GBSSI* data (Figure 3).

**Supplemental Figure S5.** Maximum likelihood phylogeny of clade AB-GBI of *Avena* inferred from nuclear *GBSSI* data (Figure 3).

**Supplemental Figure S6.** Maximum likelihood phylogeny of clade AB&A’C-GBI of *Avena* inferred from nuclear *GBSSI* data (Figure 3).

**Supplemental Figure S7.** Bayesian inference phylogeny of clade A’C-GBI of *Avena* inferred from nuclear *GBSSI* data (Figure 4).

**Supplemental Figure S8.** Bayesian inference phylogeny of clade A’C-GBII of *Avena* inferred from nuclear *GBSSI* data (Figure 4).

**Supplemental Figure S9.** Bayesian inference phylogeny of clade AB-GBI of *Avena* inferred from nuclear *GBSSI* data (Figure 4).

**Supplemental Figure S10.** Bayesian inference phylogeny of clade AB-GBII of *Avena* inferred from nuclear *GBSSI* data (Figure 4).

**Supplemental Figure S11.** Maximum likelihood phylogeny of *Avena* inferred from nuclear *gpa1* data (Figure 5).

**Supplemental Table S1.** Taxa included in this study

**Supplemental Table S2.** DNA primers and PCR parameters used for amplification and sequencing

**Supplemental Table S3.** Statistics and evolutionary models for separate data partitions

**Supplemental Table S4.** Posterior age distributions of major nodes in *Avena*

**Supplemental Table S5.** Potential paternal parents for *Avena sativa*

**
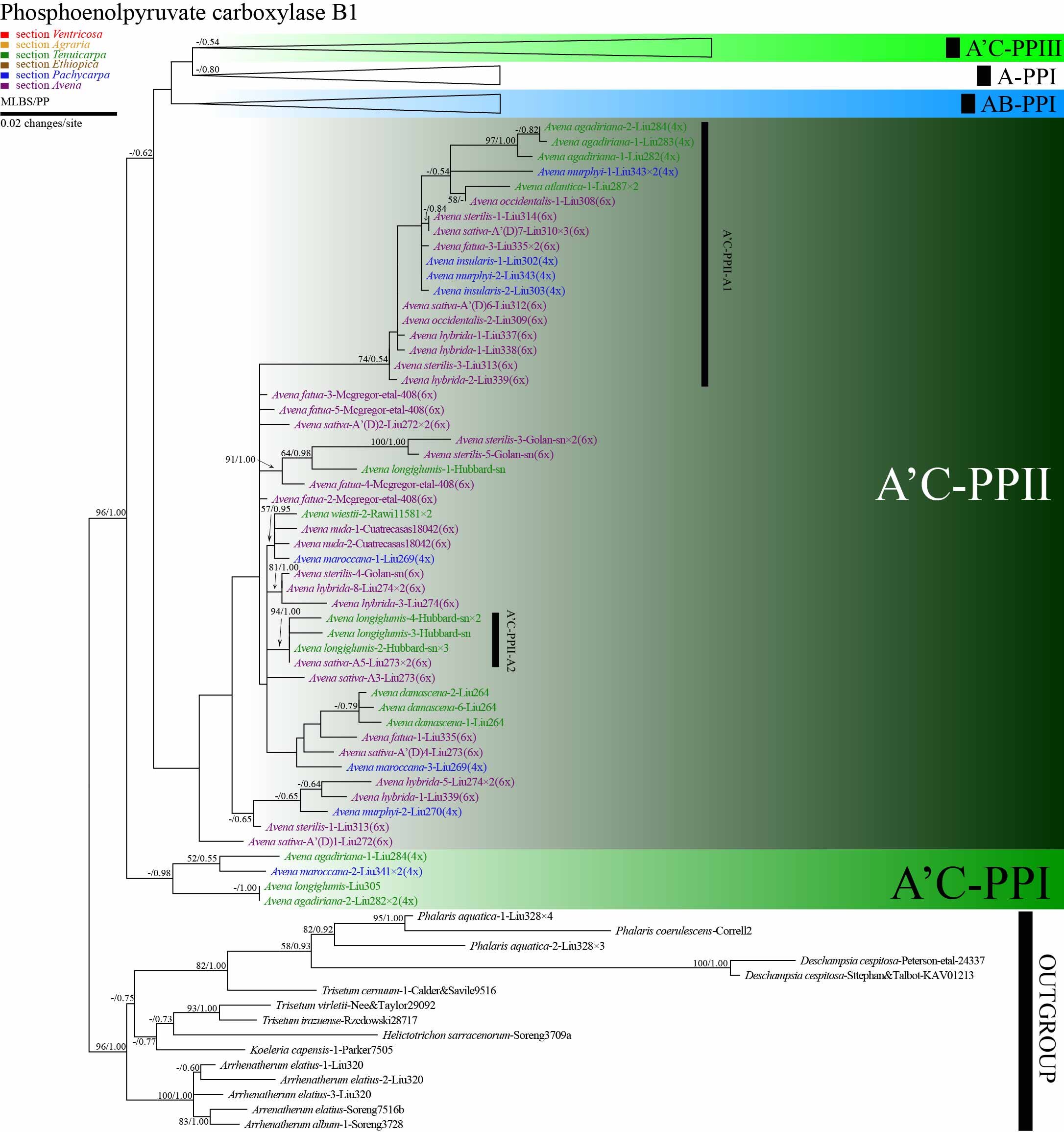
**

**Supplementary Figure S1.** Maximum likelihood phylogeny of clade A’C-PPI and node A’C-PPII of *Avena* inferred from nuclear *ppcB1* data (Figure 2). Numbers above branches are maximum likelihood bootstrap support/Bayesian posterior probability (MLBS/PP). Taxon labels are in the format: *Avena nuda*-1-Liu346×6 (6x) where *Avena nuda* indicates that the sequence belongs to the species; -1- = the first sequence cloned in Table S1 for this species; Liu346 indicates voucher; ×6 indicates we recovered 6 clones for the sequence; (6x) indicates the species is hexaploid; the absence of a mark between species name and voucher indicates that the sequence is derived from PCR-direct sequencing; the absence of a mark after voucher indicates that only one sequence for the diploid species was recovered. Coloured taxon labels correspond to sections listed at the top left corner of the figure.

**
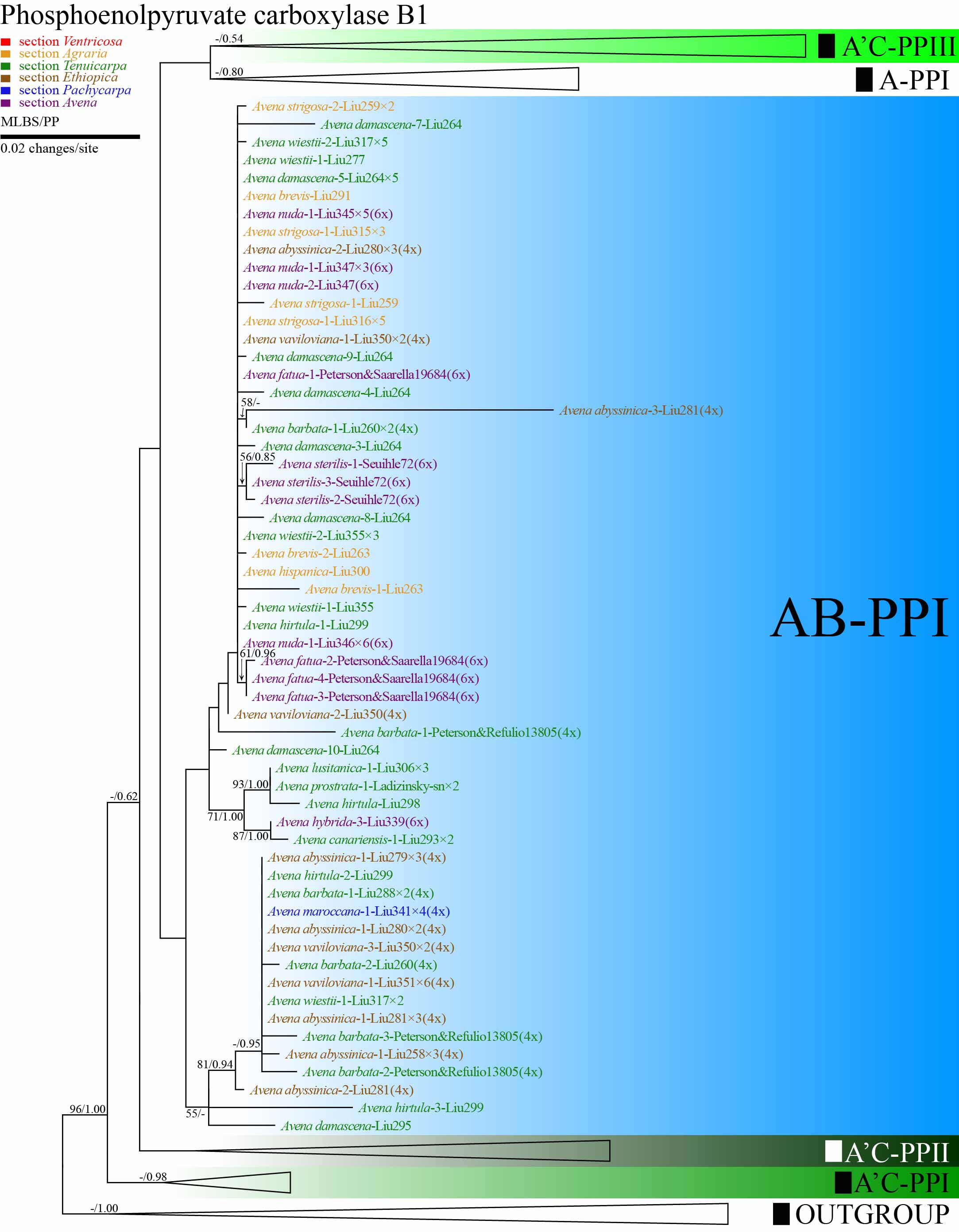
Supplementary Figure S2.** Maximum likelihood phylogeny of node AB-PPI of *Avena* inferred from nuclear *ppcB1* data (Figure 2). Numbers above branches are MLBS/PP. Taxon labels are in the same format as in Figure S1. Coloured taxon labels correspond to sections listed at the top left corner of the figure.

**
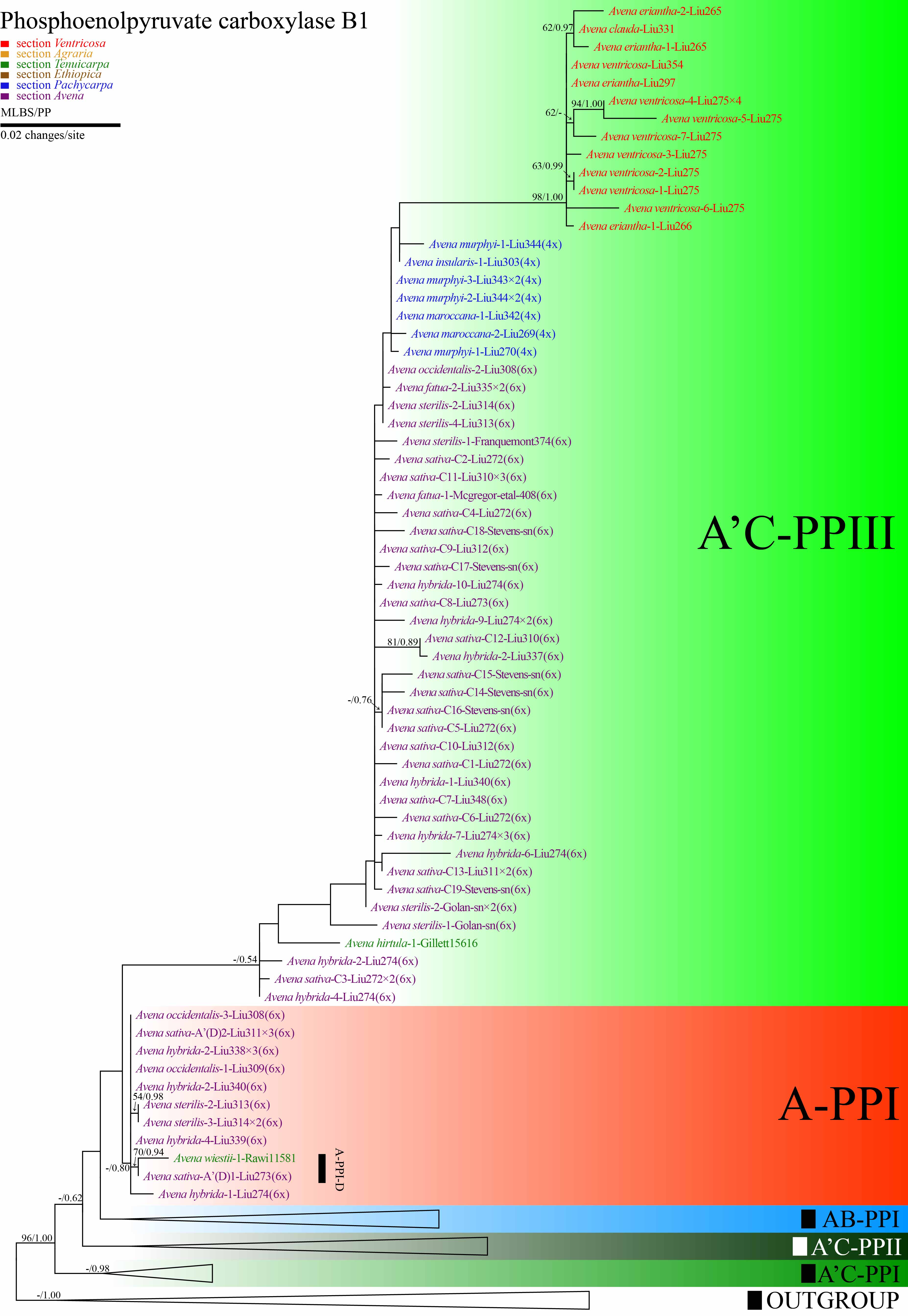
**

**Supplementary Figure S3.** Maximum likelihood phylogeny of clade A’C-PPII of *Avena* inferred from nuclear *ppcB1* data (Figure 2). Numbers above branches are MLBS/PP. Taxon labels are in the same format as in Figure S1. Coloured taxon labels correspond to sections listed at the top left corner of the figure.

**
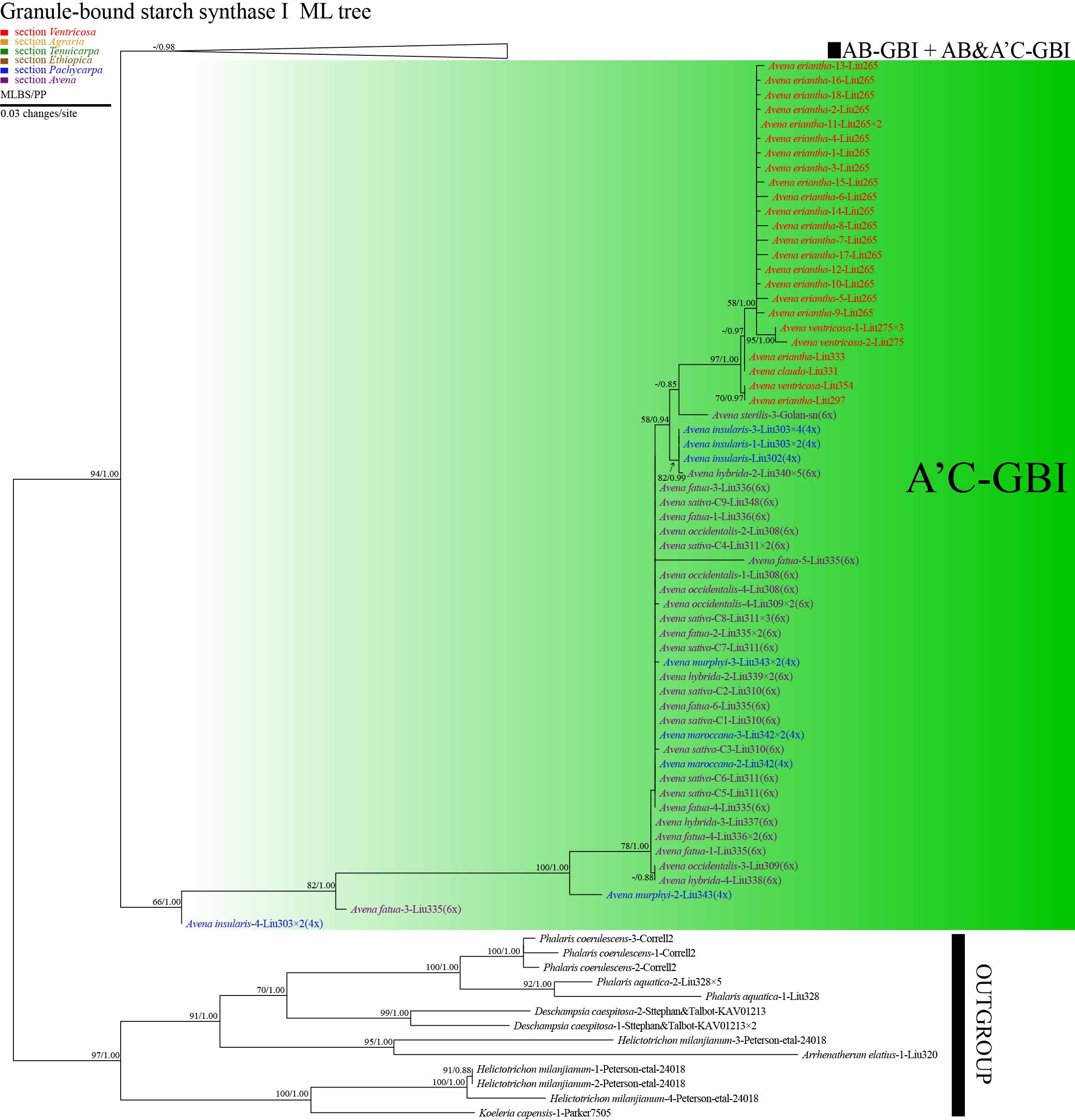
Supplementary Figure S4.** Maximum likelihood phylogeny of clade A’C-GBI of *Avena* inferred from nuclear *GBSSI* data (Figure 3). Numbers above branches are MLBS/PP. Taxon labels are in the same format as in Figure S1. Coloured taxon labels correspond to sections listed at the top left corner of the figure.

**
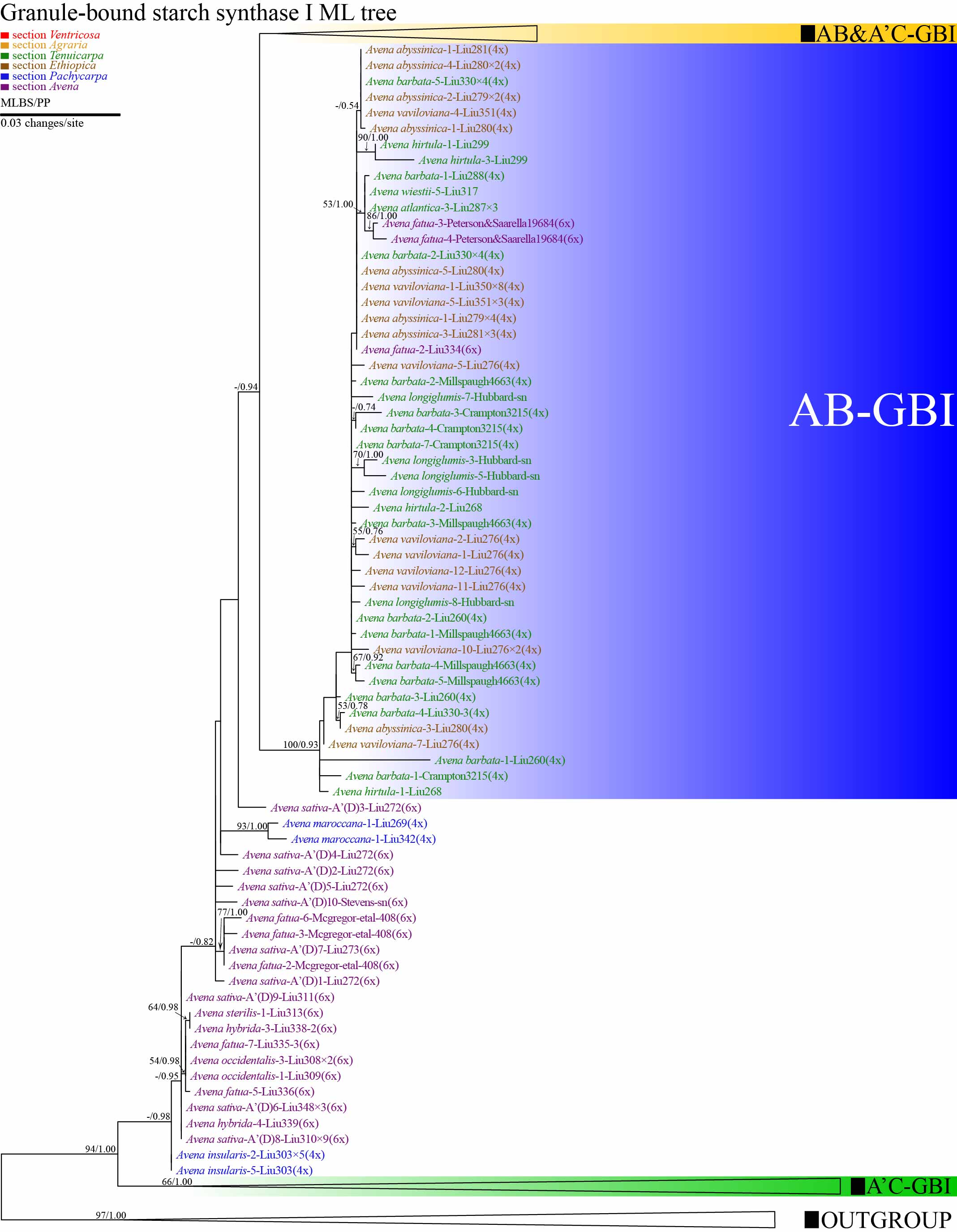
Supplementary Figure S5** Maximum likelihood phylogeny of clade AB-GBI of *Avena* inferred from nuclear *GBSSI* data (Figure 3). Numbers above branches are MLBS/PP. Taxon labels are in the same format as in Figure S1. Coloured taxon labels correspond to sections listed at the top left corner of the figure.

**
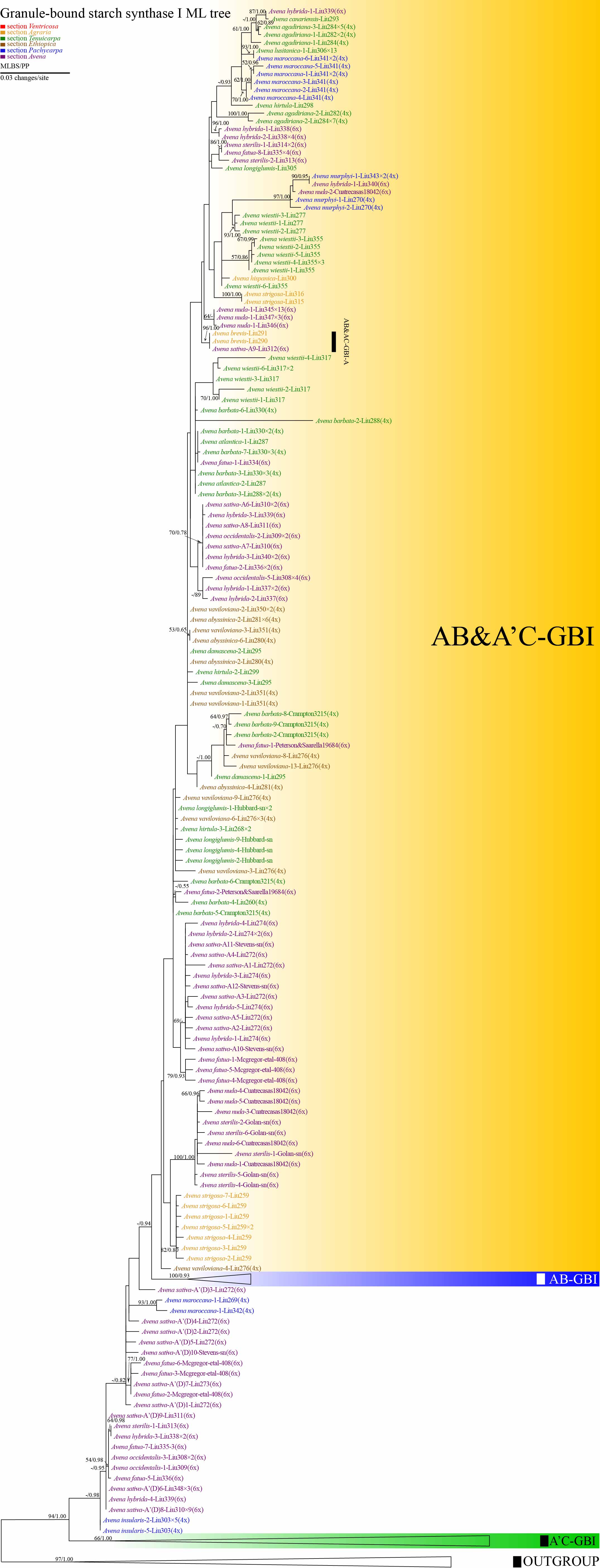
**

**Supplementary Figure S6** Maximum likelihood phylogeny of clade AB&A’C-GBI of *Avena* inferred from nuclear *GBSSI* data (Figure 3). Numbers above branches are MLBS/PP. Taxon labels are in the same format as in Figure S1. Coloured taxon labels correspond to sections listed at the top left corner of the figure.

**
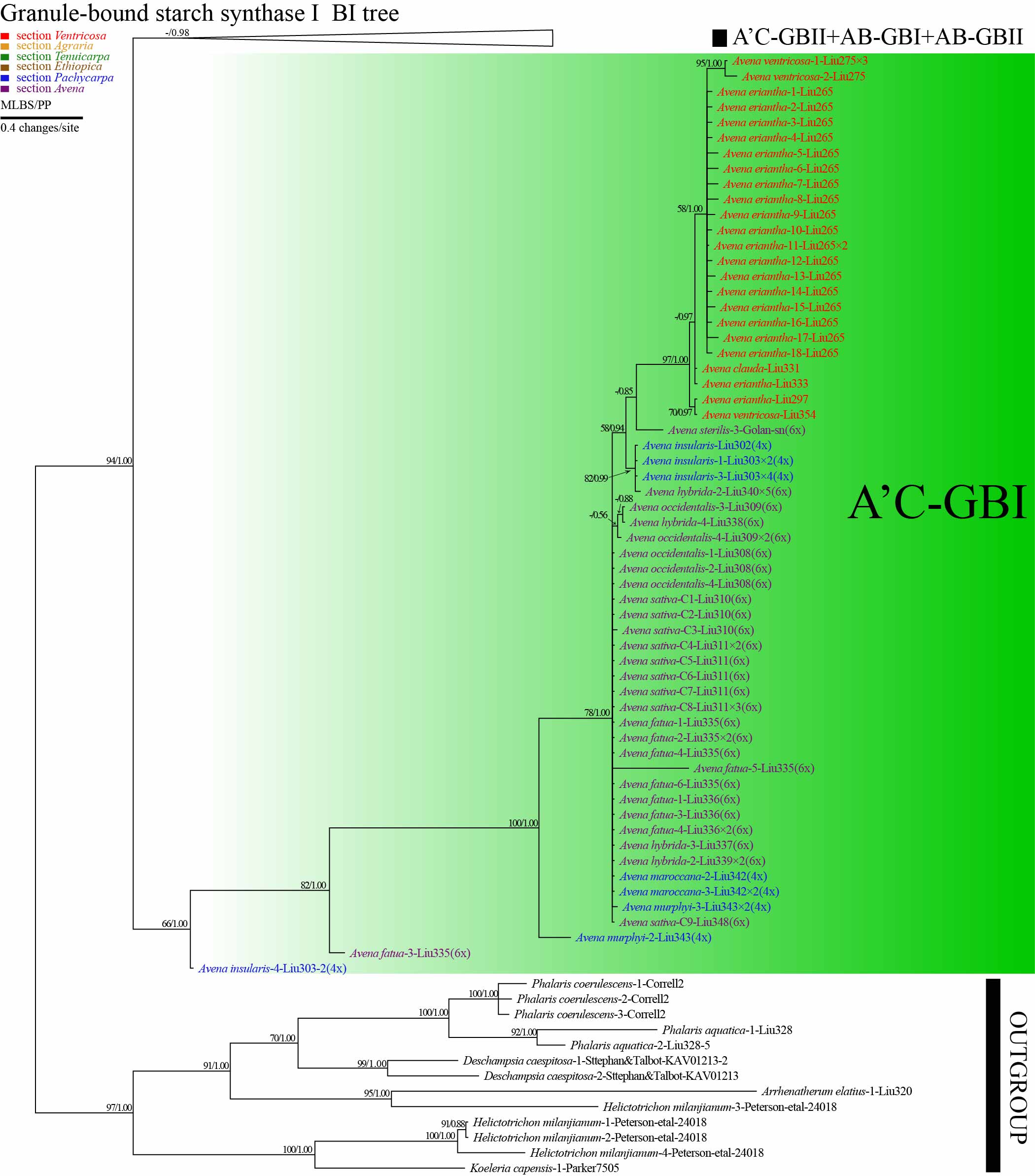
**

**Supplementary Figure S7** Bayesian inference phylogeny of clade A’C-GBI of *Avena* inferred from nuclear *GBSSI* data (Figure 4). Numbers above branches are MLBS/PP. Taxon labels are in the same format as in Figure S1. Coloured taxon labels correspond to sections listed at the top left corner of the figure.

**
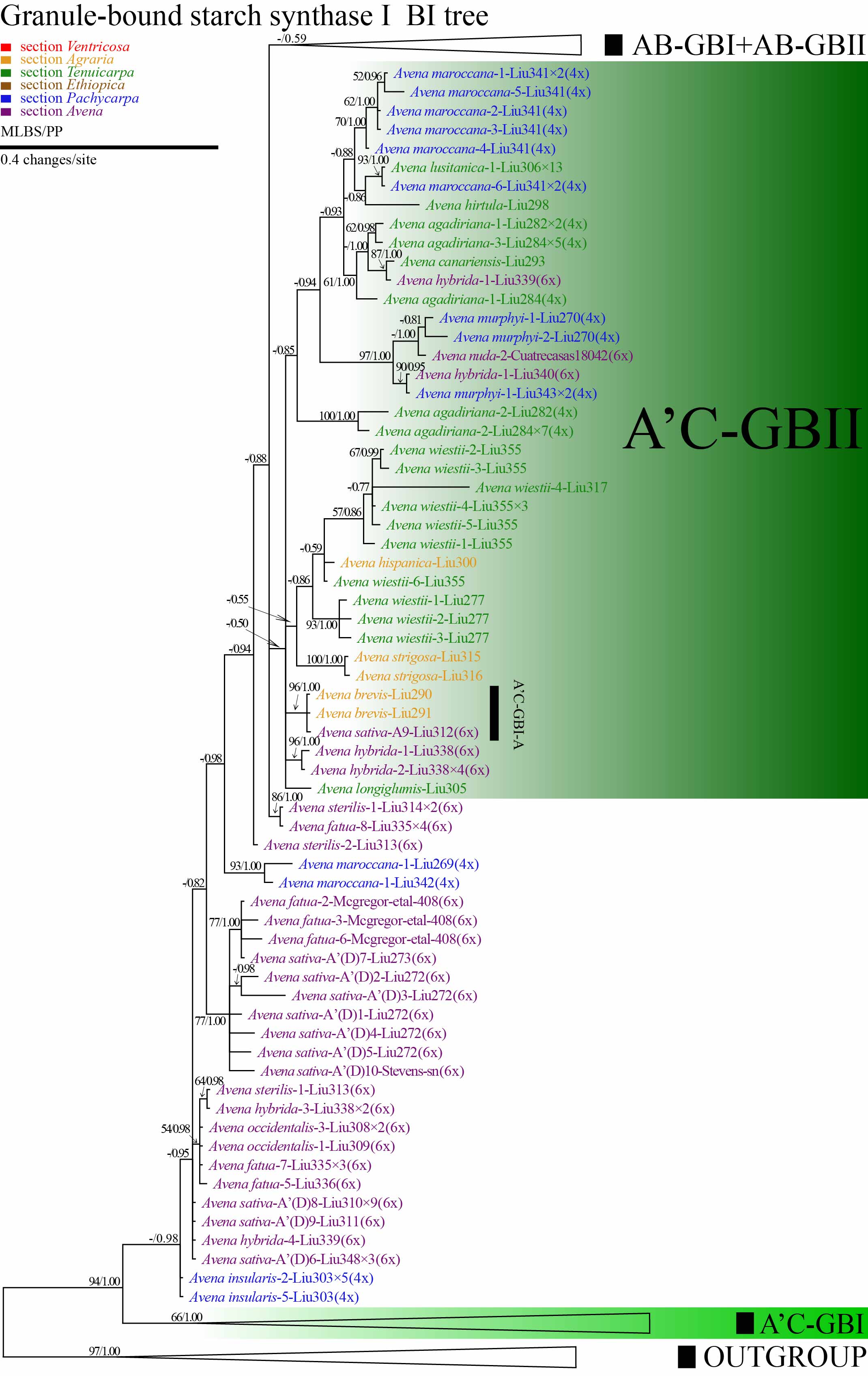
**

**Supplementary Figure S8** Bayesian inference phylogeny of clade A’C-GBII of *Avena* inferred from nuclear *GBSSI* data (Figure 4). Numbers above branches are MLBS/PP. Taxon labels are in the same format as in Figure S1. Coloured taxon labels correspond to sections listed at the top left corner of the figure.

**
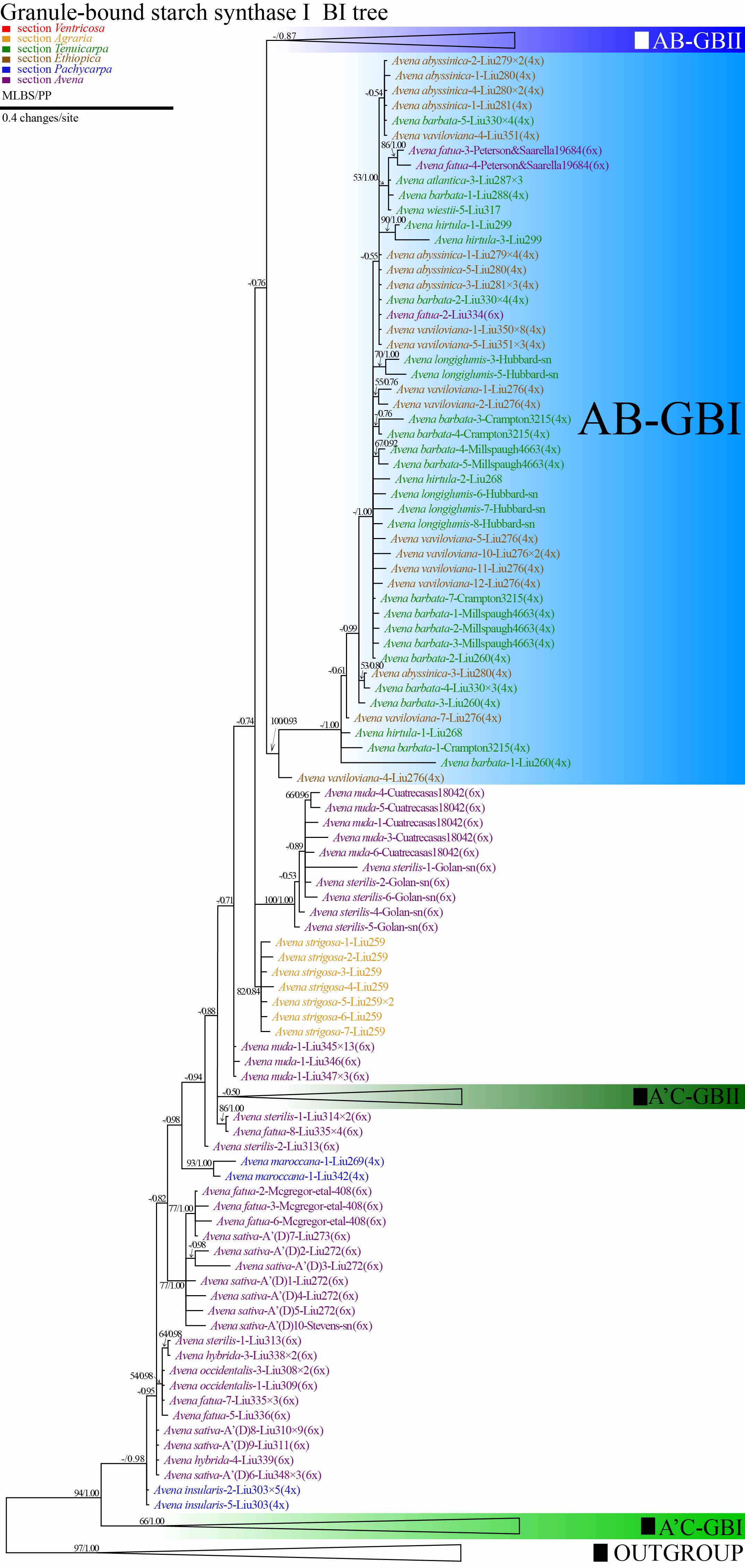
**

**Supplementary Figure S9** Bayesian inference phylogeny of clade AB-GBI of *Avena* inferred from nuclear *GBSSI* data (Figure 4). Numbers above branches are MLBS/PP. Taxon labels are in the same format as in Figure S1. Coloured taxon labels correspond to sections listed at the top left corner of the figure.

**
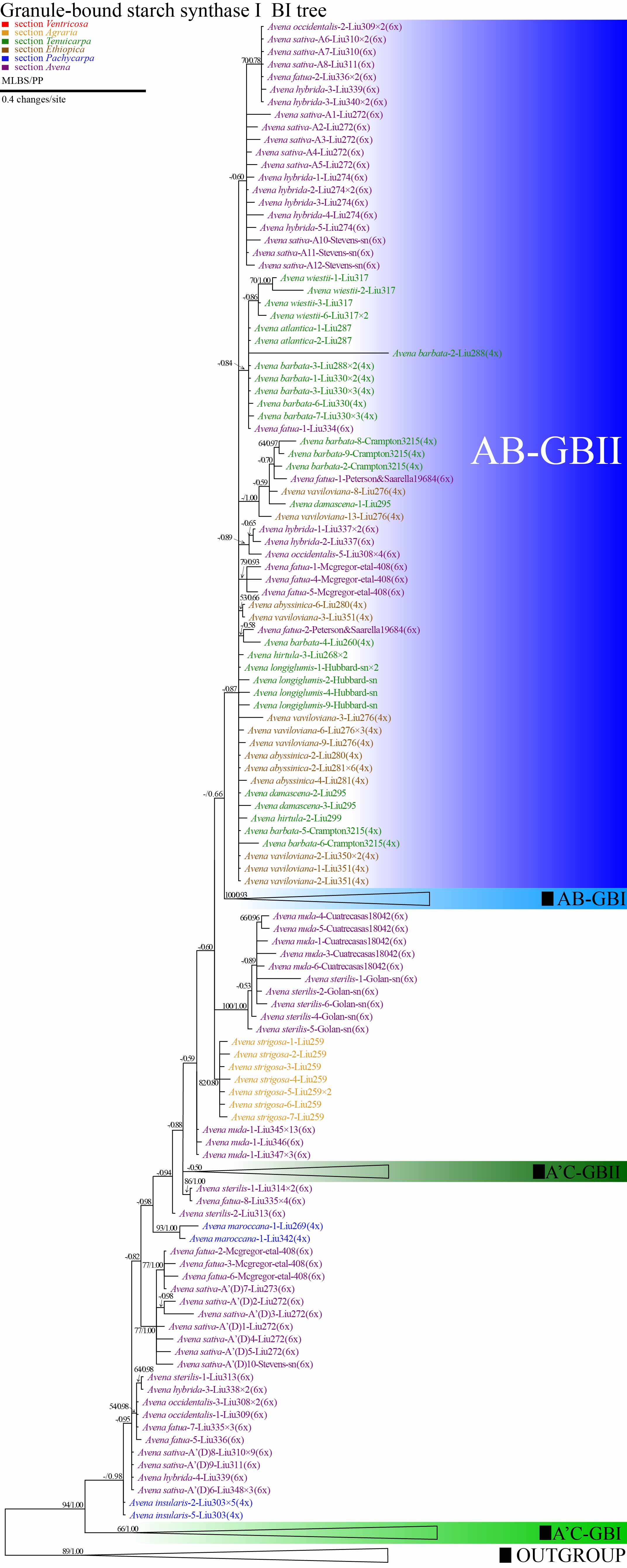
**

**Supplementary Figure S10** Bayesian inference phylogeny of clade AB-GBII of *Avena* inferred from nuclear *GBSSI* data (Figure 4). Numbers above branches are MLBS/PP. Taxon labels are in the same format as in Figure S1. Coloured taxon labels correspond to sections listed at the top left corner of the figure.

**
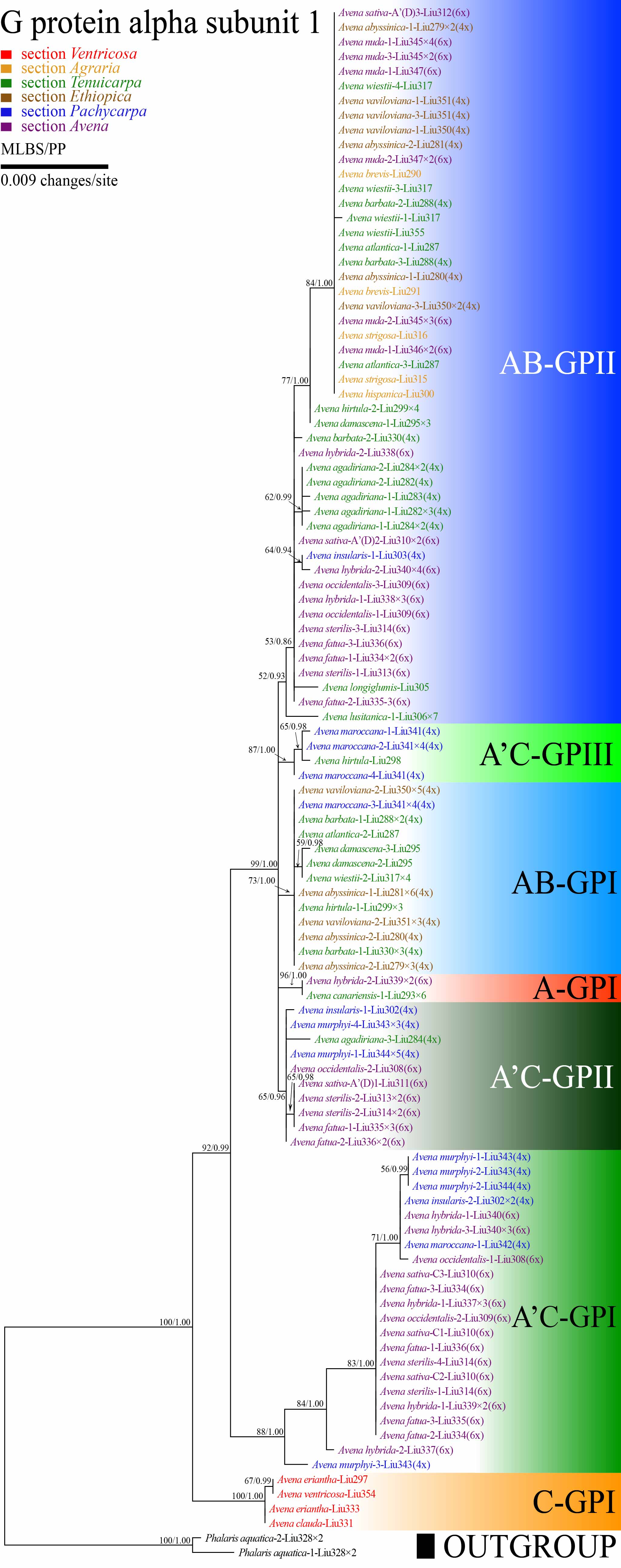
**

**Supplementary Figure S11** Maximum likelihood phylogeny of *Avena* inferred from nuclear *gpa1* data (Figure 5). Numbers above branches are MLBS/PP. Taxon labels are in the same format as in Figure S1. Coloured taxon labels correspond to sections listed at the top left corner of the figure.

# Supplementary Table S1. Taxa included in this study

| Taxa | Voucher (Source) | Country of origin | GenBank accession numbers of LCN markers (*ppc-B1*; *GBSSI*; *gpa1*) | GenBank accession numbers of chloroplastid regions (*ndhA* intron; *rpl32-trnL*; *rps16* intron) |
| --- | --- | --- | --- | --- |
| Section *Ventricosa* Baum ex Romero-Zarco | | | | |
| *A. clauda* Dur. (2*x* = 14; C_p_C_p_) | *Liu 331* (*CN 19201*; IBSC) | Canada | KT723587; KT723827; KT723996 | KT452977; KT453085; KT453177 |
| *A. eriantha* Dur. (2*x* = 14; C_p_C_p_) | *Liu 265* (*CIav 9050*; IBSC) | United Kingdom | **1 KT723655**, **2 KT723656**; **1 KT723917**, **2 KT723918**, **3 KT723919**, **4 KT723920**, **5 KT723921**, **6 KT723922**, **7 KT723923**, **8 KT723924**, **9 KT723925**, **10 KT723926**, **11 KT723927**, **12 KT723928**, **13 KT723929**, **14 KT723930**, **15 KT723931**, **16 KT723932**, **17 KT723933**, **18 KT723934**; - | KT453015; KT453034; KT453223 |
|  | *Liu 266* (*PI 657576*; IBSC) | Morocco | **1 KT723465**; -; - | KT452937; KT453035; KT453122 |
|  | *Liu 297* (*PI 657576*; IBSC) | Morocco | KT723542; KT723753; KT723959 | KT452958; KT453061; KT453151 |
|  | *Liu 333* (*CIav 9050*; IBSC) | United Kingdom | -; KT723828; KT723997 | KT452978; KT453086; KT453178 |
| *A. ventricosa* Balansa ex Coss. (2*x* = 14; CvCv) | *Liu 275* (*PI 657337*; IBSC) | Morocco | **1 KT723511**, **2 KT723512**, **3 KT723513**, **4 KT723514**, **5 KT723515**, **6 KT723516**, **7 KT723517**; **1 KT723706**, **2 KT723707**; - | KT452947; KT453044; KT453133 |
|  | *Liu 354* (*PI 657337*; IBSC) | Morocco | KT723618; KT723887; KT724039 | KT452998; KT453103; KT453197 |
| Section *Agraria* Baum | | | | |
| *A. brevis* Roth (2*x* = 14; A_s_A_s_) | *Liu 263* (*CIav 1783*; IBSC) | Germany | **1 KT723643**, **2 KT723644**;-; - | KT452990; KT453119; KT453221 |
|  | *Liu 289* (*CN 1979*; IBSC) | Canada | -; - ; - | KT452987; KT453055; KT453145 |
|  | *Liu 290* (*CN 3075*; IBSC) | Russian Federation | -; KT723747; KT723953 | KT452988; KT453057; KT453147 |
|  | *Liu 291* (*CIav 1783*; IBSC) | Germany | KT723539; KT723747; KT723954 | KT452989; KT453058; KT453148 |
| *A. hispanica* Ard. (2*x* = 14; A_s_A_s_) | *Liu 300* (*CN 25675*; IBSC) | Portugal | KT723549; KT723765; KT723963 | KT452961; KT453065; KT453156 |
| *A. strigosa* Schreb (2*x* = 14; A_s_A_s_) | *Liu 259* (*PI 401794*; IBSC) | United Kingdom | **1 KT723547**, **2 KT723548**; **1 KT723758**, **2 KT723759**, **3 KT723760**, **4 KT723761, 5 KT723762**, **6 KT723763**, **7 KT723764**; - | KT452960; -; KT453155 |
|  | *Liu 315* (*CN 21993*; IBSC) | Portugal | **1 KT723578**; KT723800; KT723986 | KT452972; KT453077; KT453169 |
|  | *Liu 316* (*CN 36500*; IBSC) | Canada | **1 KT723579**; KT723801; KT723987 | KT452973; KT453078; KT453170 |
|  | *Liu 349* (*PI 401794*; IBSC) | United Kingdom | -; - ; - | KT452995; -; KT453194 |
| Section *Tenuicarpa* Baum |  |  |  |  |
| *A. agadiriana* Baum & Fedak (4*x* = 28; AABB) | *Liu 262* (*PI 657585*; IBSC) | Morocco | -; - ; - | -; KT453118; KT453220 |
|  | *Liu 282* (*CN 25823*; IBSC) | Morocco | **1 KT723531**, **2 KT723532**; **1 KT723736**, **2 KT723737**; **1 KT723941**, **2 KT723942** | KT452953; -; KT453141 |
|  | *Liu 283* (*CN 25868*; IBSC) | Morocco | **1 KT723533**; -; **1 KT723943** | -; - ; - |
|  | *Liu 284* (*PI 657585*; IBSC) | Morocco | **1 KT723534**, **2 KT723535**; **1 KT723738**, **2 KT723739**, **3 KT723740**; **1 KT723944**, **2 KT723945**, **3 KT723946** | KT452954; KT453052; KT453142 |
| *A. atlantica* Baum & Fedak (2*x* = 14; A_s_A_s_) | *Liu 261* (*PI 657393*; IBSC) | Morocco | -; - ; - | -; KT453112; KT453212 |
|  | *Liu 287* (*PI 657393*; IBSC) | Morocco | **1 KT723536**; **1 KT723741**, **2 KT723742**, **3 KT723743**; **1 KT723947**, **2 KT723948**, **3 KT723949** | KT452955; KT453053; KT453143 |
| *A. barbata* Pott ex Link (4*x* = 28; AABB) | *Liu 260* (*PI 282723*; IBSC) | Israel | **1 KT723633**, **2 KT723634**; **1 KT723902**, **2 KT723903**, **3 KT723904**, **4 KT723905**; - | KT453016; KT453110; KT453204 |
|  | *Liu 288* (*CN 19357*; IBSC) | Iran | **1 KT723537**; **1 KT723744**, **2 KT723745**, **3 KT723746**; **1 KT723950**, **2 KT723951**, **3 KT723952** | KT453017; KT453054; KT453144 |
|  | *Liu 330* (*PI 282723*; IBSC) | Israel | **-**; **1 KT723820**, **2 KT723821**, **3 KT723822**, **4 KT723823**, **5 KT723824**, **6 KT723825**, **7 KT723826**; **1 KT723994**, **2 KT723995** | KT453018; KT453084; KT453176 |
|  | *Soderstrom 1479* (US) | Tunisia | -; - ; - | -; KT453064; KT453154 |
|  | *Peterson & Refulio 13805* (US) | Peru | **1 KT723560**, **2 KT723561**, **3 KT723562**; -; - | -; -; KT453163 |
|  | *Crampton 3215* (US) | USA | -; **1 KT723808**, **2 KT723809**, **3 KT723810**, **4 KT723811**, **5 KT723812**, **6 KT723813**, **7 KT723814**, **8 KT723815**, **9 KT723816**; - | -; KT453080; KT453172 |
|  | *Wiggins 20450* (US) | USA | -; -; - | -; KT453083; KT453175 |
|  | *Millspaugh 4663* (US) | USA | -; **1 KT723855**, **2 KT723856**, **3 KT723857**, **4 KT723858**, **5 KT723859**; - | -; KT453092; KT453184 |
| *A. canariensis* Baum & Raj & Samp (2*x* = 14; AcAc) | *Liu 293* (*CN 23021*; IBSC) | Spain | **1 KT723540**; KT723749; **1 KT723955** | KT452956; KT453059; KT453149 |
| *A. damascena* Rajah & Baum (2*x* = 14; A_d_A_d_) | *Liu 264* (*PI 657472*; IBSC) | Morocco | **1 KT723645**, **2 KT723646**, **3 KT723647**, **4 KT723648**, **5 KT723649**, **6 KT723650**, **7 KT723651**, **8 KT723652**, **9 KT723653**, **10 KT723654**; -; - | KT453014; KT453120; KT453222 |
|  | *Liu 295* (*PI 657472*; IBSC) | Morocco | KT723541; **1 KT723750**, **2 KT723751**, **3 KT723752**; **1 KT723956**, **2 KT723957**, **3 KT723958** | KT452957; KT453060; KT453150 |
| *A. hirtula* Lag. (2*x* = 14; AsAs) | *Liu 268* (*PI 657464*; IBSC) | Morocco | -; **1 KT723663**, **2 KT723664**, **3 KT723665**; - | KT452939; KT453037; KT453124 |
|  | *Liu 298* (*CN 19738*; IBSC) | Algeria | KT723543; KT723754; KT723960 | KT452959; KT453062; KT453152 |
|  | *Liu 299* (*PI 657464*; IBSC) | Morocco | **1 KT723544**, **2 KT723545**, **3 KT723546**; **1 KT723755**, **2 KT723756**, **3 KT723757**; **1 KT723961**, **2 KT723962** | KT453021; KT453063; KT453153 |
|  | *Gillett 15616* (US) | Jordan | **1 KT723538**; -; - | -; KT453056; KT453146 |
| *A. longiglumis* Dur. (2*x* = 14; AlAl) | *Liu 305* (*CN 21406*; IBSC) | Algeria | KT723553; KT723772; KT723967 | KT452964; KT453068; KT453159 |
|  | *Hubbard s.n.* (US) | United Kingdom | **1 KT723471**, **2 KT723472**, **3 KT723473**, **4 KT723474**; **1 KT723666**, **2 KT723667**, **3 KT723668**, **4 KT723669**, **5 KT723670**, **6 KT723671**, **7 KT723672**, **8 KT723673**, **9 KT723674**; - | KT452940; KT453038; KT453038 |
|  | *Soderstrom 1464* (US) | Tunisia | -; -; - | KT452950; KT453049; KT453138 |
| *A. lusitanica* (Tab. Morais) Baum (2*x* = 14; AsAs) | *Liu 306* (*CN 26251*; IBSC) | Morocco | **1 KT723554**; **1 KT723773**; **1 KT723968** | KT452965; KT453069; KT453160 |
| *A. prostrata* Ladiz. (2*x* = 14; A_p_A_p_) | *Ladizinsky s.n.* (K) | Spain | **1 KT723464**; -; - | KT452936; KT453026; KT453121 |
| *A. wiestii* Steud. (2*x* = 14; AsAs) | *Liu 277* (*PI 53626*; IBSC) | Egypt | **1 KT723518**; **1 KT723721**, **2 KT723722**, **3 KT723723**; - | KT452949; KT453046; KT453135 |
|  | *Liu 317* (*CN 19343*; IBSC) | Iran | **1 KT723580**, **2 KT723581**; **1 KT723802**, **2 KT723803**, **3 KT723804**, **4 KT723805**, **5 KT723806**, **6 KT723807**; **1 KT723988**, **2 KT723989**, **3 KT723990**, **4 KT723991** | KT452974; KT453079; KT453171 |
|  | *Liu 355* (*PI 53626*; IBSC) | Egypt | **1 KT723619**, **2 KT723620**; **1 KT723888**, **2 KT723889**, **3 KT723890**, **4 KT723891**, **5 KT723892**, **6 KT723893**; KT724040 | KT452999; KT453104; KT453198 |
|  | *Rawi 11581* (US) | Kuwait | **1 KT723523**, **2 KT723524**; -; - | -; -; - |
| Section *Ethiopica* Baum | | | | |
| *A. abyssinica* Hochst. (4*x* = 28; AABB) | *Liu 258* (*PI 58728*; IBSC) | Ethiopia | **1 KT723505**; -; - | KT452945; KT453043; KT453131 |
|  | *Liu 279* (*CN 3971*; IBSC) | Canada | **1 KT723525**; **1 KT723724**, **2 KT723725**; **1 KT723935**, **2 KT723936** | -; -; - |
|  | *Liu 280* (*CN 22051*; IBSC) | Ethiopia | **1 KT723526**, **2 KT723527**; **1 KT723726**, **2 KT723727**, **3 KT723728**, **4 KT723729**, **5 KT723730**, **6 KT723731**; **1 KT723937**, **2 KT723938** | KT452951; KT453050; KT453139 |
|  | *Liu 281* (*PI 58728*; IBSC) | Ethiopia | **1 KT723528**, **2 KT723529**, **3 KT723530**; **1 KT723732**, **2 KT723733**, **3 KT723734**, **4 KT723735**; **1 KT723939**, **2 KT723940** | KT452952; KT453051; KT453140 |
| *A. vaviloviana* (Malz.) Mordv (4*x* = 28; AABB) | *Liu 276* (*PI 412766*; IBSC) | Ethiopia | -; **1 KT723708**, **2 KT723709**, **3 KT723710**, **4 KT723711**, **5 KT723712**, **6 KT723713**, **7 KT723714**, **8 KT723715**, **9 KT723716**, **10 KT723717**, **11 KT723718**, **12 KT723719**, **13 KT723720**; - | KT452948; KT453045; KT453134 |
|  | *Liu 350* (*CN 22004*; IBSC) | Ethiopia | **1 KT723614**, **2 KT723615**, **3 KT723616**; **1 KT723880**, **2 KT723881**; **1 KT724033**, **2 KT724034**, **3 KT724035** | KT452996; KT453101; KT453195 |
|  | *Liu 351* (*PI 412766*; IBSC) | Ethiopia | **1 KT723617**; **1 KT723882**, **2 KT723883**, **3 KT723884**, **4 KT723885**, **5 KT723886**; **1 KT724036**, **2 KT724037**, **3 KT724038** | KT452997; KT453102; KT453196 |
| Section *Pachycarpa* Baum | | | | |
| *A. insularis* Ladiz. [4*x* = 28; AACC(DDCC)] | *Liu 302* (*CN 19178*; IBSC) | Italy | **1 KT723550**; KT723766; **1 KT723964**, **2 KT723965** | KT452962; KT453066; KT453157 |
|  | *Liu 303* (*CN 108634*; IBSC) | Tunisia | **1 KT723551**, **2 KT723552**; **1 KT723767**, **2 KT723768**, **3 KT723769**, **4 KT723770**, **5 KT723771**; **1 KT723966** | KT452963; KT453067; KT453158 |
| *A. maroccana* Grand. [4*x* = 28; AACC(DDCC)] | *Liu 269* (*CIav 8330*; IBSC) | Morocco | **1 KT723475**, **2 KT723476**, **3 KT723477**; **1 KT723675**; - | KT453019; KT453039; KT453126 |
|  | *Liu 341* (*CN 21862*; IBSC) | Morocco | **1 KT723601**, **2 KT723602**; **1 KT723863**, **2 KT723864**, **3 KT723865**, **4 KT723866**, **5 KT723867**, **6 KT723868**; **1 KT724016**, **2 KT724017**, **3 KT724018**, **4 KT724019** | KT453022; KT453094; KT453186 |
|  | *Liu 342* (*CIav 8330*; IBSC) | Morocco | **1 KT723603**; **1 KT723869**, **2 KT723870**, **3 KT723871**; **1 KT724020** | KT453023; KT453095; KT453187 |
| *A. murphyi* Ladiz. [4*x* = 28; AACC(DDCC)] | *Liu 270* (*PI 657606*; IBSC) | Morocco | **1 KT723478**, **2 KT723479**; **1 KT723676**, **2 KT723677**; - | KT452941; KT453040; KT453127 |
|  | *Liu 343* (*CN 21989*; IBSC) | Spain | **1 KT723604**, **2 KT723605**, **3 KT723606**; **1 KT723872**, **2 KT723873**, **3 KT723874**; **1 KT724021**, **2 KT724022**, **3 KT724023**, **4 KT724024** | KT452986; KT453096; KT453188 |
|  | *Liu 344* (*PI 657606*; IBSC) | Morocco | **1 KT723607**, **2 KT723608**;-; **1 KT724025**, **2 KT724026** | KT452991; KT453097; KT453189 |
| Section *Avena* | | | | |
| *A. fatua* L. (6*x* = 42; AACCDD) | *Liu 334* (*CN 3214*; IBSC) | Australia | -; **1 KT723829**, **2 KT723830**; **1 KT723998**, **2 KT723999**, **3 KT724000** | KT452979; -; KT453179 |
|  | *Liu 335* (*CN 3228*; IBSC) | Australia | **1 KT723588**, **2 KT723589**, **3 KT723590**; **1 KT723831**, **2 KT723832**, **3 KT723833**, **4 KT723834**, **5 KT723835**, **6 KT723836**, **7 KT723837**, **8 KT723838**; **1 KT724001**, **2 KT724002**, **3 KT724003** | KT452980; KT453087; KT453180 |
|  | *Liu 336* (*PI 544659*; IBSC) | USA | -; **1 KT723839**, **2 KT723840**, **3 KT723841**, **4 KT723842**, **5 KT723843**; **1 KT724004**, **2 KT724005**, **3 KT724006** | KT452981; KT453088; KT453181 |
|  | *Mcgregor et al. 408* (US) | Mexico | **1 KT723466**, **2 KT723467**, **3 KT723468**, **4 KT723469**, **5 KT723470**; **1 KT723657**, **2 KT723658**, **3 KT723659**, **4 KT723660**, **5 KT723661**, **6 KT723662**; - | KT452938; KT453036; KT453123 |
|  | *Peterson & Saarella 19684* (US) | USA | **1 KT723627**, **2 KT723628**, **3 KT723629**, **4 KT723630**; **1 KT723898**, **2 KT723899**, **3 KT723900**, **4 KT723901**; - | KT453001; KT453106; KT453200 |
| *A. hybrida* Peterm. (6*x* = 42; AACCDD) | *Liu 274* (*PI 458784*; IBSC) | United Kingdom | **1 KT723495**, **2 KT723496**, **3 KT723497**, **4 KT723498**, **5 KT723499**, **6 KT723500**, **7 KT723501**, **8 KT723502**, **9 KT723503**, **10 KT723500**; **1 KT723695**, **2 KT723696**, **3 KT723697**, **4 KT723698**, **5 KT723699**; - | KT452944; -; KT453130 |
|  | *Liu 337* (*CN 24885*; IBSC) | Iran | **1 KT723591**, **2 KT723592**; **1 KT723844**, **2 KT723845**, **3 KT723846**; **1 KT724007**, **2 KT724008** | KT452982; KT453089; KT453182 |
|  | *Liu 338* (*CN 24926*; IBSC) | Iran | **1 KT723593**, **2 KT723594**; **1 KT723847**, **2 KT723848**, **3 KT723849**, **4 KT723850**; **1 KT724009**, **2 KT724010** | KT452983; KT453090; - |
|  | *Liu 339* (*PI 458778*; IBSC) | United Kingdom | **1 KT723595**, **2 KT723596**, **3 KT723597**, **4 KT723598**; **1 KT723851**, **2 KT723852**, **3 KT723853**, **4 KT723854**; **1 KT724011**, **2 KT724012** | KT452984; KT453091; KT453183 |
|  | *Liu 340* (*PI 458784*; IBSC) | United Kingdom | **1 KT723599**, **2 KT723600**; **1 KT723860**, **2 KT723861**, **3 KT723862**; **1 KT724013**, **2 KT724014**, **3 KT724015** | KT452985; KT453093; KT453185 |
| *A. nuda* L. (6*x* = 42; AACCDD) | *Liu 345* (*CN 79351*; IBSC) | Netherlands | **1 KT723609**; **1 KT723875**; **1 KT724027**, **2 KT724028**, **3 KT724029** | KT452992; KT453098; KT453190 |
|  | *Liu 346* (*CN 79386*; IBSC) | Germany | **1 KT723610**; **1 KT723876**; **1 KT724030** | KT452993; -; KT453191 |
|  | *Liu 347* (*CIav 9008*; IBSC) | Czechoslovakia | **1 KT723611**, **2 KT723612**; **1 KT723877**; **1 KT724031**, **2 KT724032** | KT452994; KT453099; KT453192 |
|  | *Cuatrecasas 18042* (US) | Colombia | **1 KT723480**, **2 KT723481**; **1 KT723678**, **2 KT723679**, **3 KT723680**, **4 KT723681**, **5 KT723682**, **6 KT723683**; - | KT452942; KT453041; KT453128 |
| *A. occidentalis* Dur. (6*x* = 42; AACCDD) | *Liu 308* (*CN 4538*; IBSC) | Spain | **1 KT723555**, **2 KT723556**, **3 KT723557**; **1 KT723774**, **2 KT723775**, **3 KT723776**, **4 KT723777**, **5 KT723778**; **1 KT723969**, **2 KT723970** | KT452966; KT453070; KT453161 |
|  | *Liu 309* (*CN 21473*; IBSC) | Greece | **1 KT723558**, **2 KT723559**; **1 KT723779**, **2 KT723780**, **3 KT723781**, **4 KT723782**; **1 KT723971**, **2 KT723972**, **3 KT723973** | KT452967; KT453071; KT453162 |
| *A. sativa* L. (6*x* = 42; AACCDD) | *Liu 272* (*PI 401777*; IBSC) | Poland | **A’ (D)1 KT723486**, **A’ (D)2 KT723489**, **C1 KT723482**, **C2 KT723483**, **C3 KT723484**, C**4 KT723485**, **C5 KT723487**, **C6 KT723488**; **A1 KT723684**, **A2 KT723686**, **A3 KT723688**, **A4 KT723689**, **A5 KT723690**, **A’(D)1 KT723685**, **A’(D)2 KT723687**, **A’(D)3 KT723691**, **A’(D)4 KT723692**, **A’(D)5 KT723693**; - | KT452943; KT453042; KT453129 |
|  | *Liu 273* (*PI 51385*; IBSC) | Spain | **A3 KT723490**, **A’ (D)4 KT723493**, **A5 KT723494**, **C8 KT723491**, **A’(D)1 KT723492**; **A’(D)7 KT723694**; - | -; -; - |
|  | *Liu 348* (*PI 401777*; IBSC) | Poland | **C7 KT723613**; **C9 KT723879**, **A’(D)6 KT723878**; - | KT453024; KT453100; KT453193 |
|  | *Liu 312* (*PI 51385*; IBSC) | Spain | **A’ (D)6 KT723568**, **C9 KT723569**, **C10 KT723570**; A9 KT723796; A’(D)3 KT723979 | KT452969; KT453074; KT453166 |
|  | *Liu 310* (*CN 1876*; IBSC) | Canada | **A’ (D)7 KT723565**, **C11 KT723563**, **C12 KT723564**; **A6 KT723783**, **A7 KT723788**, **C1 KT723784**, **C2 KT723785**, **C3 KT723787**, **A’(D)8 KT723786**; **A’(D)2 KT723977**, **C1 KT723974**, **C2 KT723975**, **C3 KT723976** | KT452968; KT453072; KT453164 |
|  | *Liu 311* (*CN 18136*; IBSC) | Canada | **C13 KT723567**, **A’(D)2 KT723566**; **A8 KT723794**, **C4 KT723790**, **C5 KT723791**, **C6 KT723792**, **C7 KT723793**, **C8 KT723795**, **A’(D)9 KT723789**; **A’(D)1 KT723978** | KT453020; KT453073; KT453165 |
|  | *Stevens s.n.* (US) | USA | **C14 KT723621**, **C15 KT723622**, **C16 KT723623**, **C17 KT723624**, **C18 KT723625**, **C19 KT723626**; **A10 KT723895**, **A11 KT723896**, **A12 KT723897**, **A’(D)10 KT723894**; - | KT453000; KT453105; KT453199 |
| *A. sterilis* L. (6*x* = 42; AACCDD) | *Liu 313* (*CN 3253*; IBSC) | Australia | **1 KT723571**, **2 KT723572**, **3 KT723573**, **4 KT723574**; **1 KT723797**, **2 KT723798**; **1 KT723980**, **2 KT723981** | KT453166; KT453075; KT453167 |
|  | *Liu 314* (*CN 3375*; IBSC) | Australia | **1 KT723575**, **2 KT723576**, **3 KT723577**; **1 KT723799**; **1 KT723982**, **2 KT723983**, **3 KT723984**, **4 KT723985** | KT452971; KT453076; KT453168 |
|  | *Golan s.n.* (US) | Palaestinae | **1 KT723506**, **2 KT723507**, **3 KT723508**, **4 KT723509**, **5 KT723510**; **1 KT723700**, **2 KT723701**, **3 KT723702**, **4 KT723703**, **5 KT723704**, **6 KT723705**; - | KT452946; -; KT453132 |
|  | *Seuihle 72* (US) | Jordan | **1 KT723519**, **2 KT723520**, **3 KT723521**; -; - | -; KT453047; KT453136 |
|  | *Franquemont 374* (US) | Peru | **1 KT723522**; -; - | -; KT453048; KT453137 |
| Outgroup |  |  |  |  |
| *Arrhenatherum* *album* (Vahl) Clayton (2*x* = 10,14) | *Soreng 3728* (US) | Spain | **1 KT723631**; -; - | KT453002; KT453107; KT453201 |
| *Arrhenatherum elatius* (L.) P. Beauv. ex J. Presl & C. Presl (4*x* = 28) | *Liu 320* (*PI 249687*; US) | Spain | **1 KT723582**, **2 KT723583**, **3 KT723584**; **1 KT723817**; - | KT452975; KT453081; KT453173 |
|  | *Soreng 7516b* (US) | Greece | KT723632; -; - | -; KT453108; KT453202 |
| *Briza minor* L. (2*x* = 14) | *Peterson et al. 9283* (US) | Ecuador | -; -; - | KT453003; KT453109; KT453203 |
| *Briza maxima* L. (2*x* = 14) | *Beetle R605* (US) | Portugal | -; -; - | -; KT453111; KT453205 |
| *Deschampsia cespitosa* (L.) P. Beauv. (2*x* = 26) | *Sttephan & Talbot KAV01213* (US) | USA | KT723635; **1 KT723906**, **2 KT723907**; - | -; -; - |
|  | *Peterson et al. 24337* (US) | Tanzania | KT723636; -; - | KT453025; KT453028; KT453206 |
| *Helictotrichon milanjianum* (Rendle) C.E. Hubb. (2*x* = 26) | *Peterson et al. 24018* (US) | Tanzania | -; **1 KT723908**, **2 KT723909**, **3 KT723910**, **4 KT723911**; - | KT453005; KT453029; KT453208 |
|  | *Peterson et al. 24034* (US) | Tanzania | -; -; - | KT453007; KT453031; KT453210 |
|  | *Peterson et al. 24122* (US) | Tanzania | -; -; - | KT453008; -; KT453211 |
| *Helictotrichon pallens* (Link) J.M. Couderc & Guédès (2*x* = 26) | *Peterson & Ollgaard 253* (US) | Denmark | -; -; - | KT453004; -; KT453207 |
| *Helictotrichon sarracenorum* (Gand.) Holub. (2*x* = 14) | *Soreng 3709a* (US) | Spain | KT723637; -; - | KT453006; KT453030; KT453209 |
| *Koeleria capensis* Nees (2*x* = 14) | *Peterson et al. 24336* (US) | USA | -; -; - | KT453010; KT453032; KT453214 |
|  | *Parker 7505* (US) | USA | **1 KT723638**; **1 KT72 3912**; - | KT453009; KT453113; KT453213 |
| *Phalaris aquatica* L. (2*x* = 28) | *Liu 328* (*PI 598922*; US) | Italy | **1 KT723585**, **2 KT723586**; **1 KT723818**, **2 KT723819**; **1 KT723992**, **2 KT723993** | KT452976; KT453082; KT453174 |
| *Phalaris angusta* Nees ex Trin. (2*x* = 14) | *Scur 654* (US) | Brazil | -; -; - | KT453013; KT453117; KT453219 |
| *Phalaris coerulescens* Desf. (2*x* = 14) | *Correll 2* (US) | Australia | KT723642; **1 KT723914**, **2 KT723915**, **3 KT723916**; - | -; KT453116; KT453218 |
| *Trisetum cernuum* Trin. (4*x* = 28) | *Calder & Savile 9516* (US) | Canada | **1 KT723639**; -; - | -; KT453033; KT453215 |
| *Trisetum irazuense* (Kuntze) Hitchc. (4*x* = 28) | *Rzedowski 28717* (US) | Mexico | KT723641; **1 KT723913**; - | KT453012; KT453115; KT453217 |
| *Trisetum virletii* E. Fourn. (4*x* = 28) | *Nee & Taylor 29092* (US) | Mexico | KT723640; -; - | KT453011; KT453114; KT453216 |

Taxa: Chromosome numbers are based on http://mobot.mobot.org/W3T/Search/ipcn2.html; Genome assignment is based on Lin & Liu^5^ and Nikoloudakis & Katsiotis^30^. Voucher (Source): CN, Plant Gene Resources at Saskatchewan, Canada; ILRI, International Livestock Research Institute at Addis Ababa, Ethiopia; PI or CIav, Germplasm Resources Information Network of United States Department of Agriculture at Beltsville, USA; IBSC, South China Botanical Garden Herbarium; K, Royal Botanic Gardens, Kew; US, United States National Herbarium; GenBank accession numbers of LCN markers (*ppc-B1*; *GBSSI*; *gpa1*) followed by sequence number (Prefix “A” or “B” or “A’(D)” indicates A- or B- or A’(D)-type homoeologue for polyploid species); interrupted line indicates unavailable sequence; Cloned sequences are labelled in bold.

# Supplementary Table S2. Primers and PCR parameters used for amplification and sequencing. Chromosomal locations of nuclear genes are based on rice (*Oryza sativa* L.)

| **Region** | **Location** | **Primers** | **Sequence (5’–3’)** | **PCR parameters** | **Reference** |
| --- | --- | --- | --- | --- | --- |
| *PpcB1* | Chromosome 1 | *PpcB1*-8F | AAG GCC CAG GAG GAG ATC GTG G | 95°C/3 min; 16 × (94°C/20 s; 65°C /40 s, -1°C/cycle; 72°C/90 s), 21 × (94°C/20 s; 50°C/40 s; 72°C/90 s); 72°C/5 min | This study |
|  |  | *PpcB1*-9R | CAG CCG CTG CCT CAG GTA CGG GT |  |  |
| *GBSSI* | Chromosome 6 | *GBSSI* 9F | ATC GTC AAC GGC ATG GAC GTC | The same as above | This study |
|  |  | *GBSSI* 14R | CAC GTC CTC CCA GTT CTT GGC |  |  |
| *gpa1* | Chromosome 5 | *gpa1*10F | GAG GAG RAA GTG GAT TCA TCT | The same as above | This study |
|  |  | *gpa1*12R | ACC TCC TGT TTG YCA KGT GC |  |  |
| *ndhA* intron | Plastid | *ndhA* intronF | CGC TAT TYC AAA ACC GTA CRT | 95°C/5 min; 36 × (94°C/30 s; 53°C/60 s; 72°C/120 s); 72°C/8 min | This study |
|  |  | *ndhA* intronR | CAA TAT CTC TAC GTG TGA TTC G |  |  |
| *rpl32-trnL* | Plastid | *rpl32*F | CAG TTC CAA AAA AAC GTA CTT C | The same as above | Liu *et al*., 2014^31^ |
|  |  | *rpl32*-trnL  ^(UAG)^R | CTG CTT CCT AAG AGC AGC GT |  |  |
| *rps16* intron | Plastid | *rps16*F | TGT GGT ARA AAG CAA C | The same as above | Liu *et al.*, 2014^31^ |
|  |  | *rps16*R | AAC ATC WAT TGC AAS GAT TCG ATA |  |  |

**Supplementary Table S3. Statistics and evolutionary models for separate data partitions. SL, aligned sequence length; GC, guanine and cytosine; PIC, parsimony informative characters; Ti/Tv, transition/transversion ratio; CI, consistency index excluding uninformative characters; RI, retention index**

| **Partition** | **No. of Sequences** | **SL** | **GC%** | **PIC** | **PIC/SL** | **Ti/Tv** | **CI** | **RI** | **Best-fit model** |
| --- | --- | --- | --- | --- | --- | --- | --- | --- | --- |
| *ppcB1* | 193 | 1017 | 0.6226 | 220 | 0.2163 | 1.1290 | 0.2676 | 0.5333 | GTR + I + G |
| *GBSSI* | 277 | 1352 | 0.5662 | 434 | 0.3210 | 1.2274 | 0.4308 | 0.8864 | TrN + G |
| *gpa1* | 106 | 1034 | 0.3652 | 137 | 0.1321 | 1.5681 | 0.9227 | 0.9849 | TVM + G |
| cpDNA | 288 | 2819 | 0.3013 | 232 | 0.0823 | 0.9281 | 0.8187 | 0.9139 | TVM + I + G |

# Supplementary Table S4. Posterior age distributions of major lineages in *Avena*. Lineage number in accordance with those in Figure 6; Lineage age is given by the mean age and the 95% highest posterior density (HPD) intervals in brackets; NA, not available

| Lineage | Number | Stem age (Mya) | Crown age (Mya) |
| --- | --- | --- | --- |
| *Avena* | 1 | 25.57 (NA) | 20.04 (3.56–35.06) |
| C-NRR | 2 | 20.04 (3.56–35.06) | 10.71 (1.62–20.25) |
| A’C-NRR + AB-NRR | 3 | 20.04 (3.56–35.06) | 14.54 (2.68–25.02) |
| A’C-NRR | 4 | 14.54 (2.68–25.02) | 12.24 (NA) |
| The *A. sativa* lineage 5 | 5 | 3.82 (NA) | 2.43 (NA) |
| The *A. satica* lineage 6 | 6 | 4.77 (NA) | 2.46 (NA) |
| The *A. sativa* lineage 7 | 7 | 4.69 (NA) | 2.97 (NA) |
| AB-NRR | 8 | 14.54 (2.68–25.02) | 10.88 (NA) |

# Supplementary Table S5. Potential paternal parents for *Avena sativa*. Bold species, strongly supported in present study (MLBS/PP); Underlined species, supported in previous studies^13,16,18,21,22^; Bold and underlined species, supported in present and previous studies

| Species | 2X (ML/BI) | | 4X (ML/BI) | |
| --- | --- | --- | --- | --- |
|  | CC genome | AA genome | AABB genome | A’A’CC genome |
| *Avena sativa* | ***A. clauda***^[18]^(100/1.00)  ***A. eriantha*** (100/1.00)  ***A. ventricosa***^[13]^(100/1.00) | *A. atlantica* (84/1.00)  ***A. brevis*** (95/1.00)  *A. damascena* (77/1.00)  *A. hirtula*^[16]^(77/1.00)  *A. hispanica* (84/1.00)  ***A. longiglumis***^[13]^(94/1.00)  *A. strigosa*^[16]^(84/1.00)  *A. wiestii*^[16]^(84/1.00) | *A. abyssinica* (84/1.00)  *A. barbata* (84/1.00)  *A. vaviloviana* (84/1.00) | *A. insularis*^[21]^(83/1.00)  *A. maroccana*^[22]^(83/1.00)  ***A. murphyi***^[22]^(100/1.00) |
