# Supplementary figures and images for: Unraveling the evolutionary dynamics of ancient and recent polypoidization events in Avena (Poaceae)
Source: Sci Rep. 2017 Feb 3;7:41944. doi: 10.1038/srep41944 (PMC5291219; doi:10.1038/srep41944)

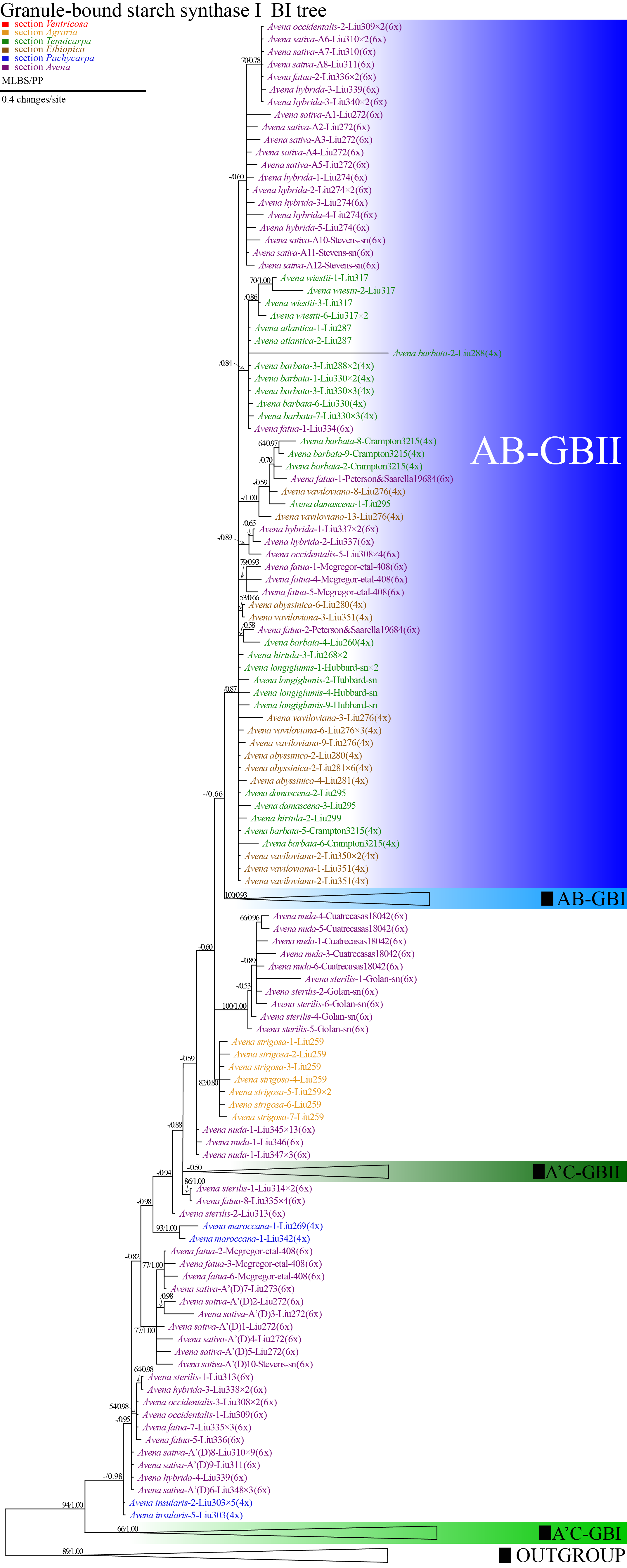

Supplement: Supplementary Figs. S1-S11 [file srep41944-s2.zip › Fig. S10AB-GBII.tif]

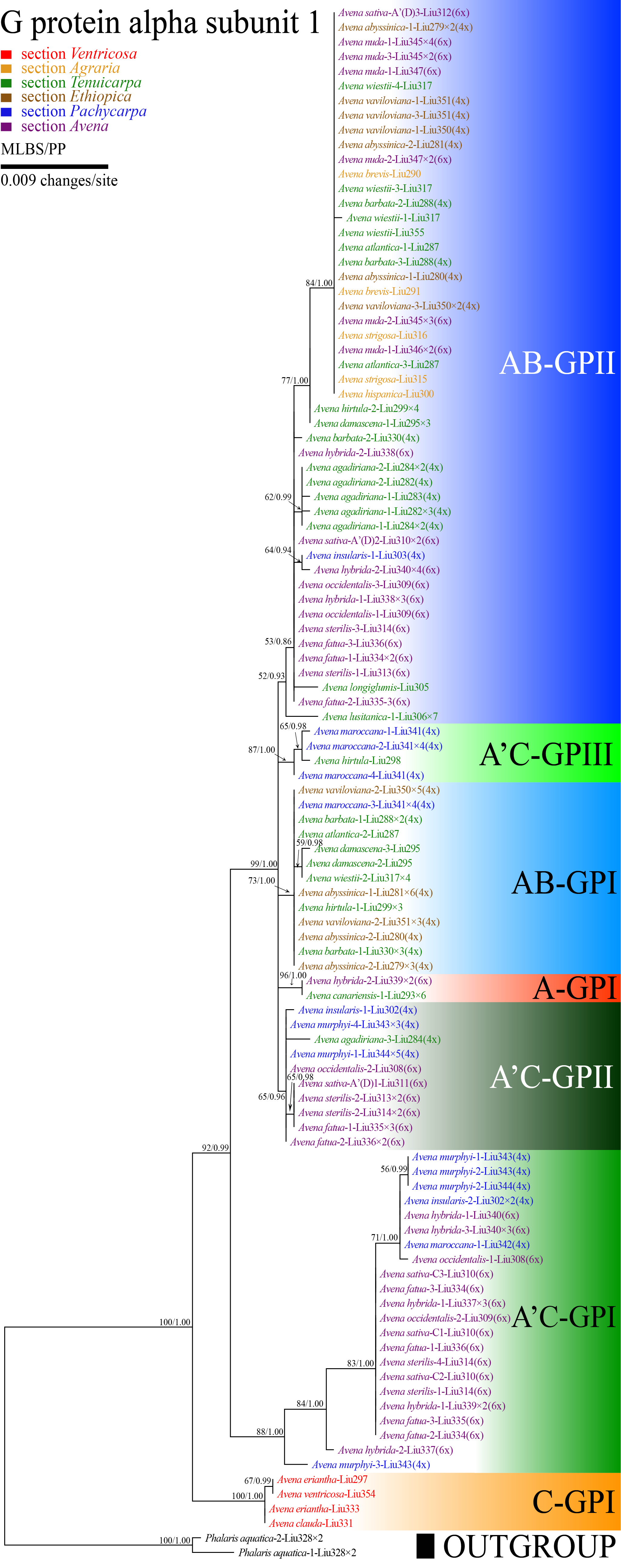

Supplement: Supplementary Figs. S1-S11 [file srep41944-s2.zip › Fig. S11-GPA1.tif]

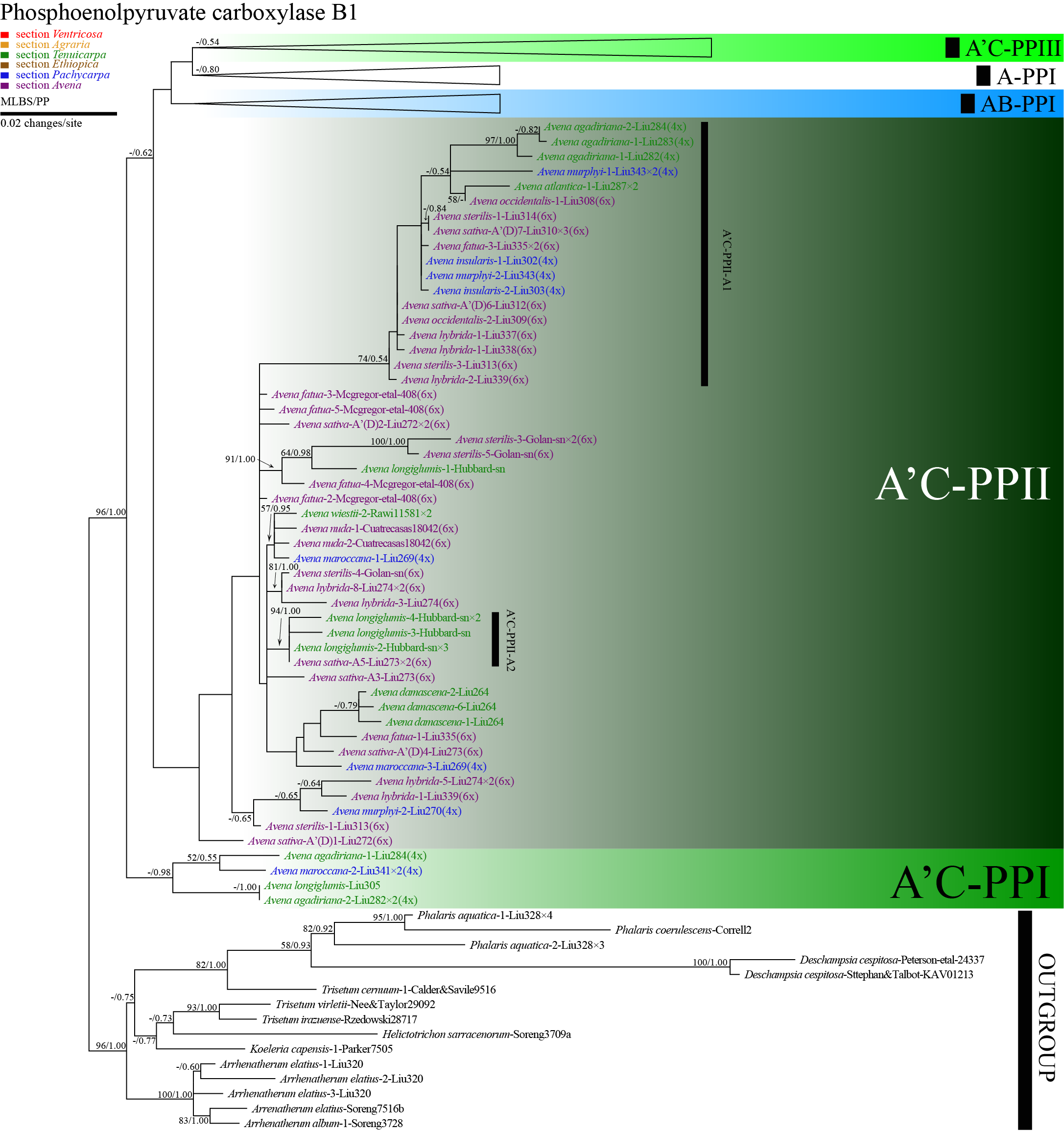

Supplement: Supplementary Figs. S1-S11 [file srep41944-s2.zip › Fig. S1A'C-PPII.tif]

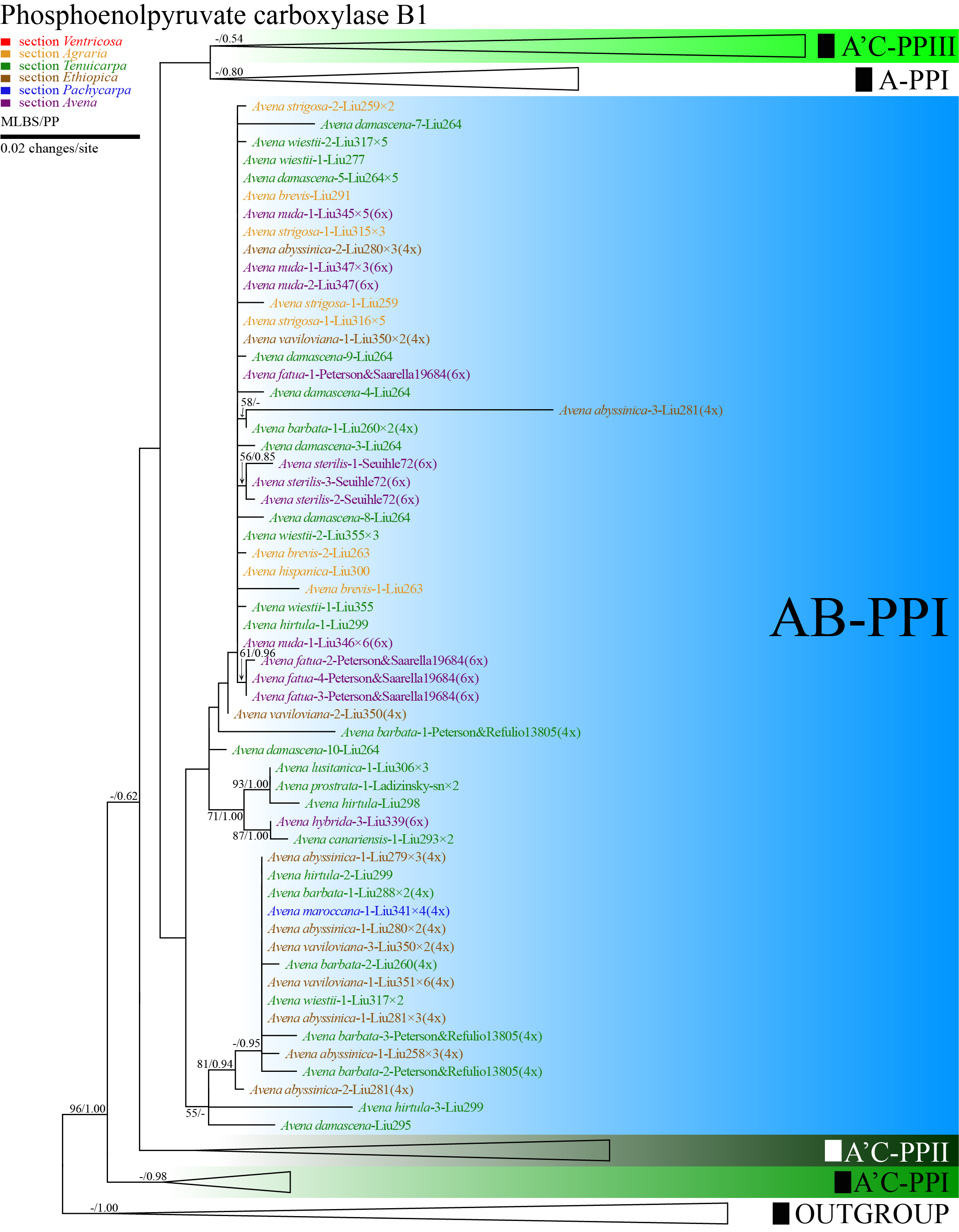

Supplement: Supplementary Figs. S1-S11 [file srep41944-s2.zip › Fig. S2AB-PPI.tif]

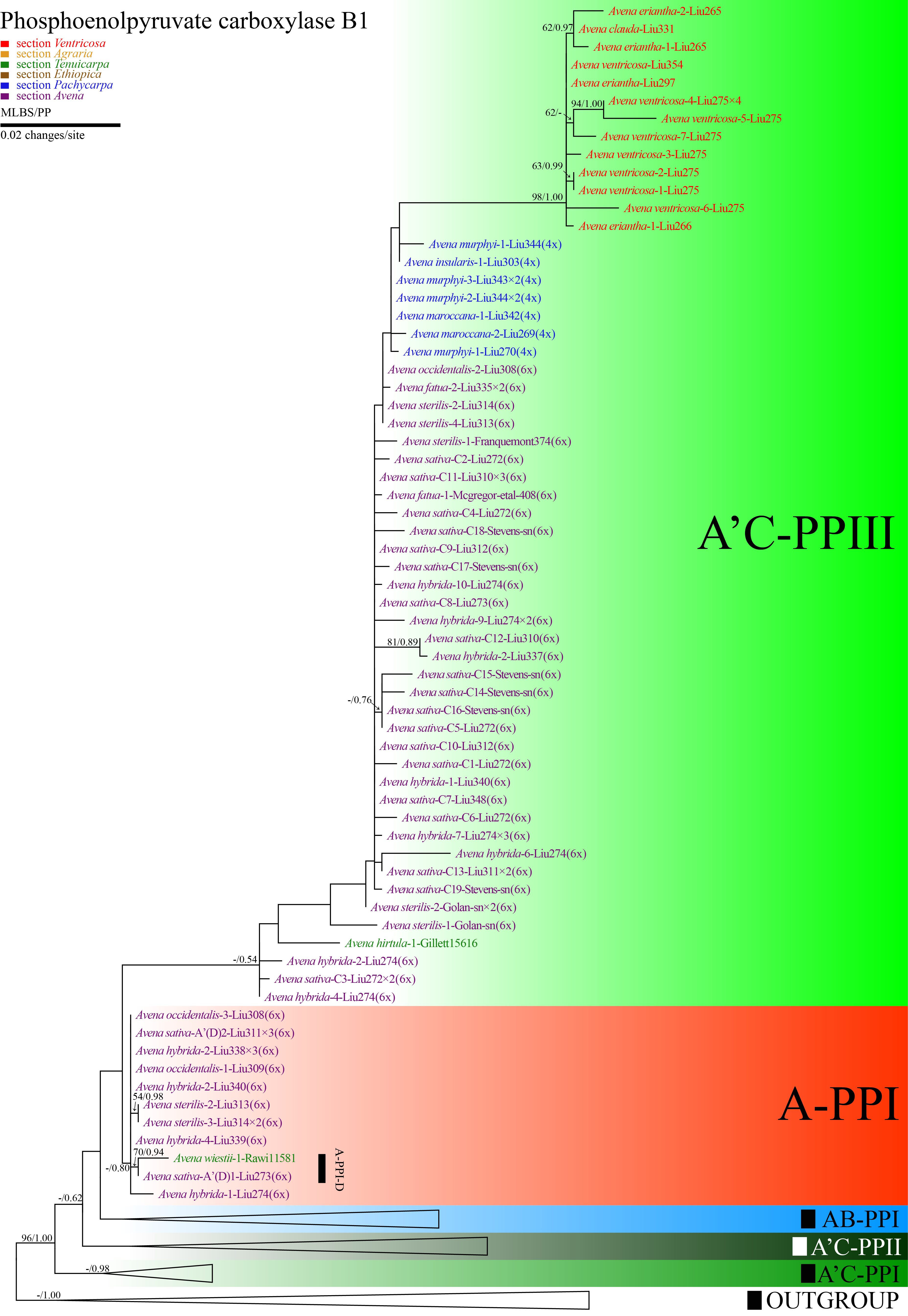

Supplement: Supplementary Figs. S1-S11 [file srep41944-s2.zip › Fig. S3A'C-PPIII.tif]

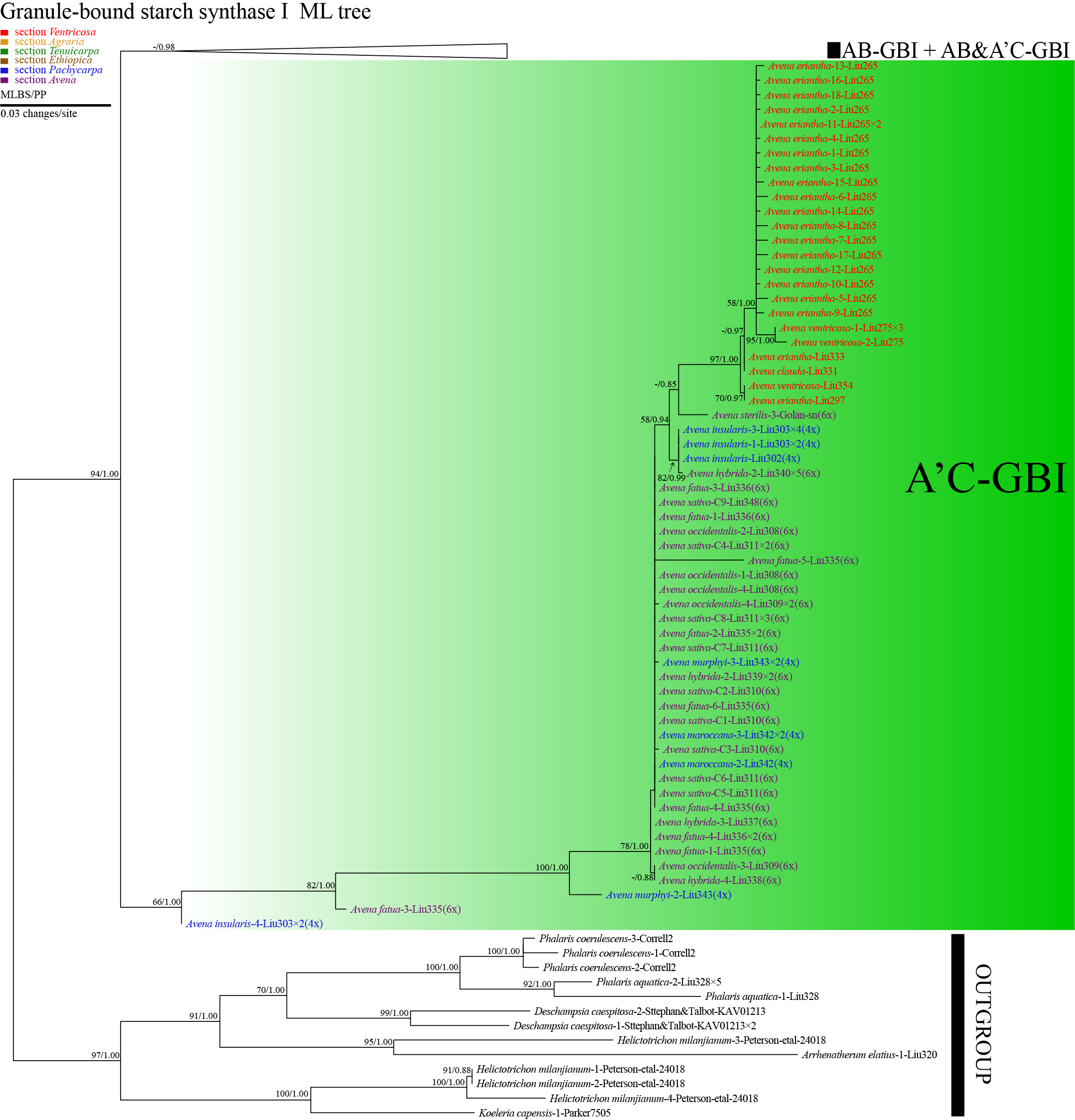

Supplement: Supplementary Figs. S1-S11 [file srep41944-s2.zip › Fig. S4A'C-GBI.tif]

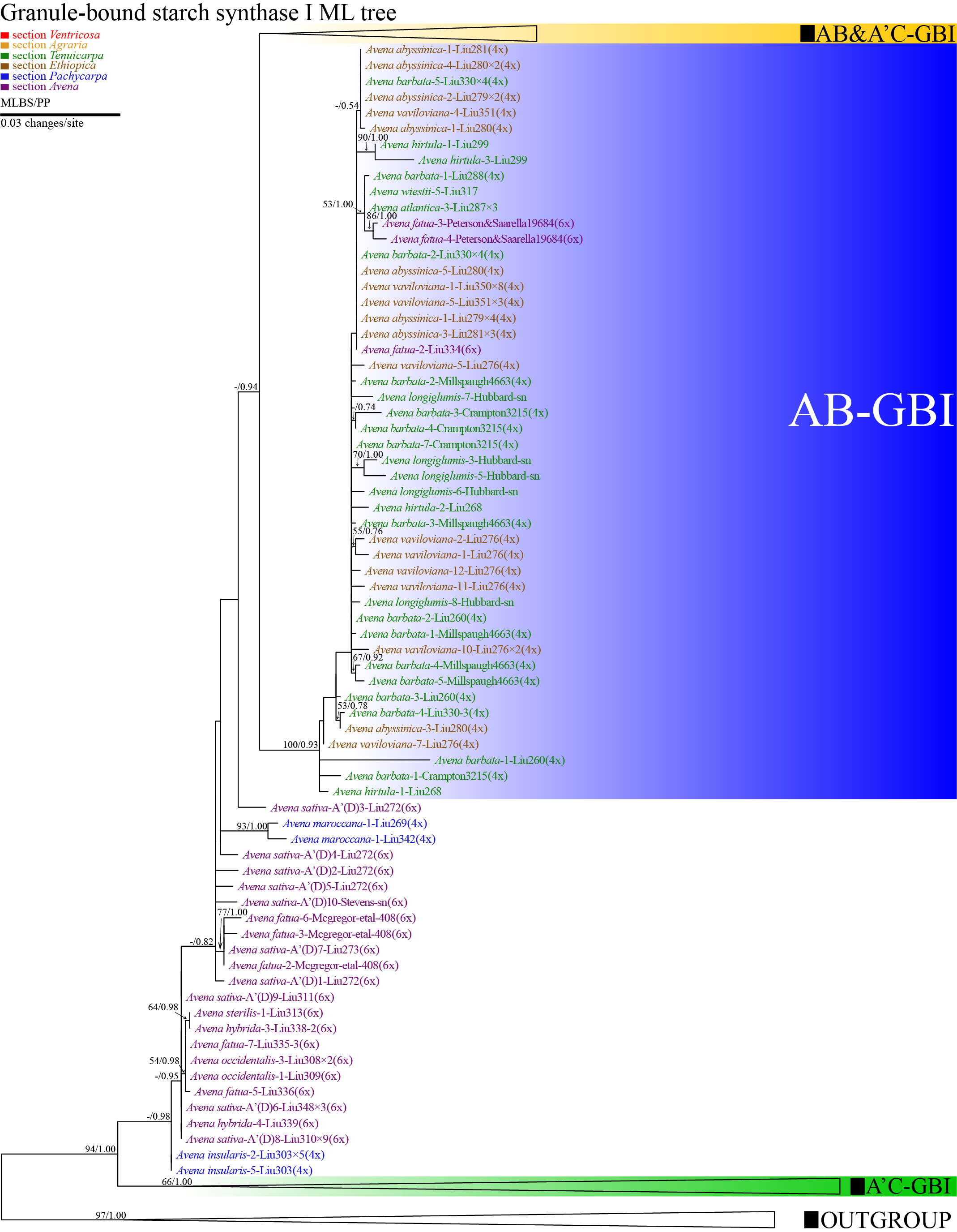

Supplement: Supplementary Figs. S1-S11 [file srep41944-s2.zip › Fig. S5AB-GBI.tif]

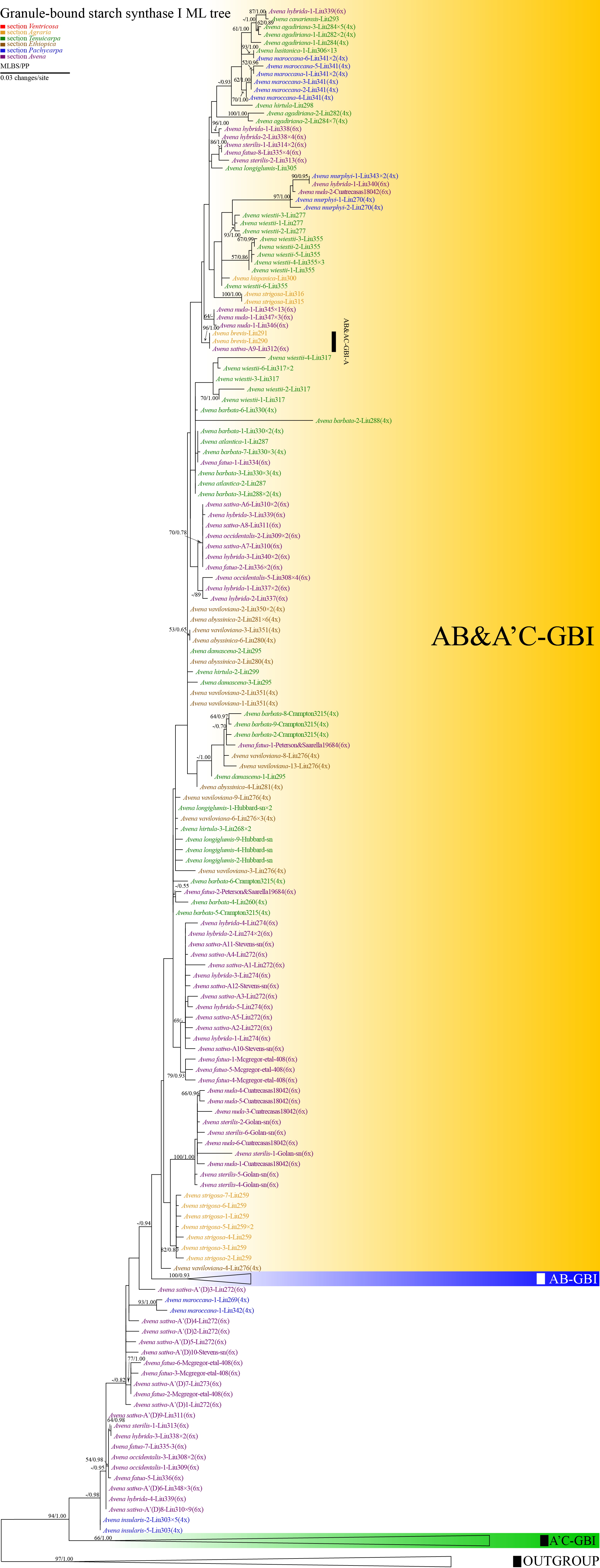

Supplement: Supplementary Figs. S1-S11 [file srep41944-s2.zip › Fig. S6AB&A'C-GBI.tif]

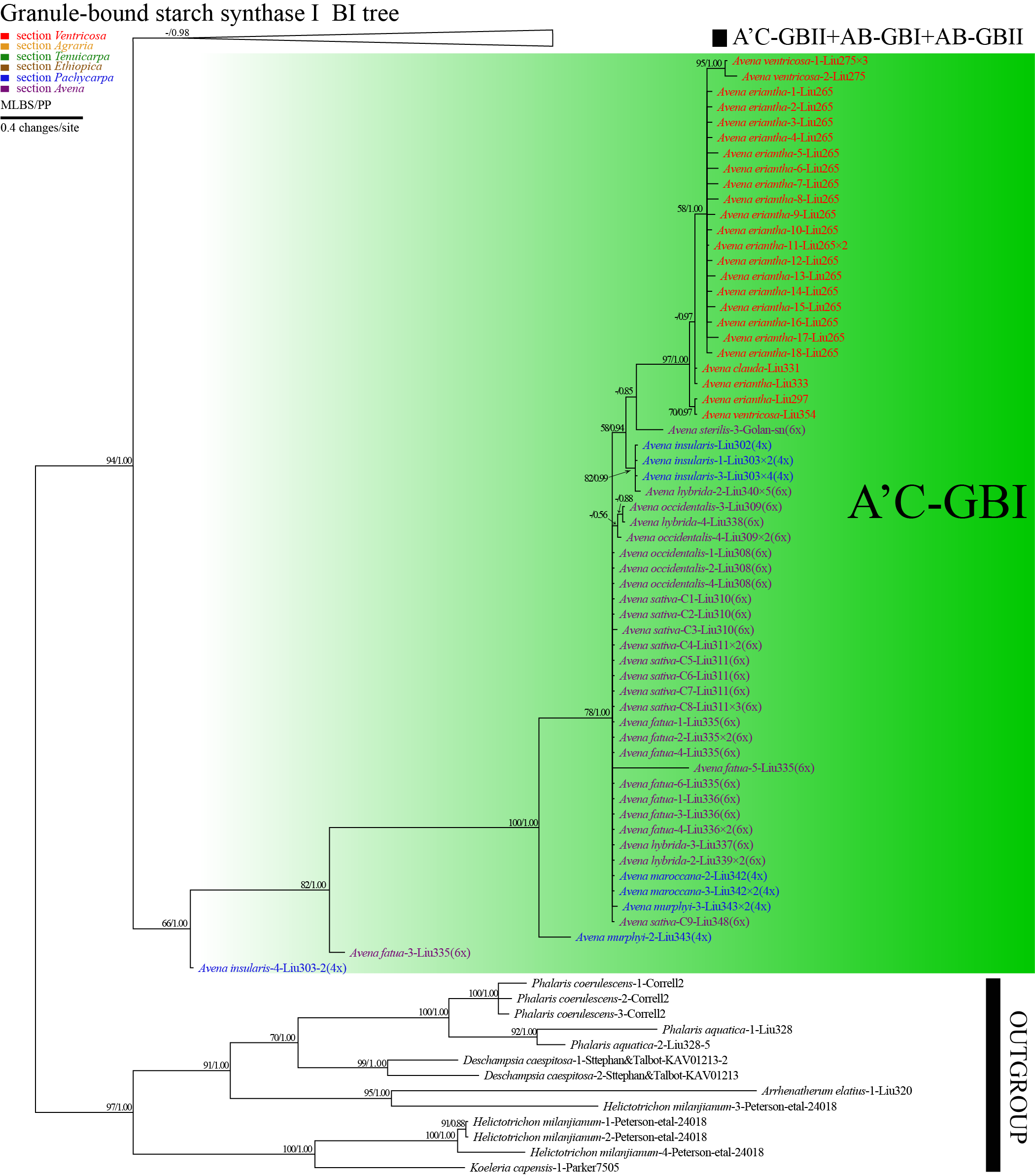

Supplement: Supplementary Figs. S1-S11 [file srep41944-s2.zip › Fig. S7A'C-GBI.tif]

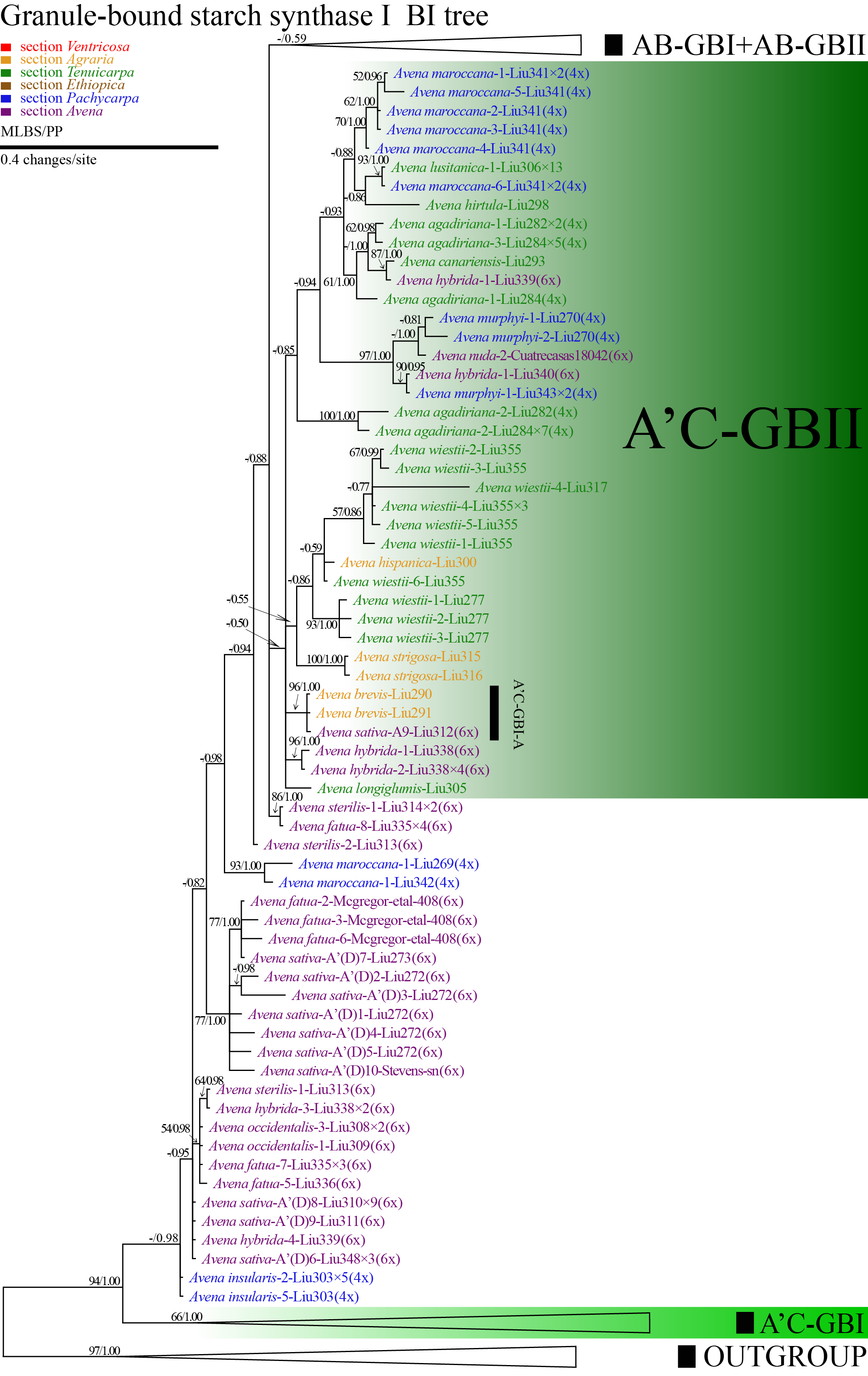

Supplement: Supplementary Figs. S1-S11 [file srep41944-s2.zip › Fig. S8A'C-GBII.tif]

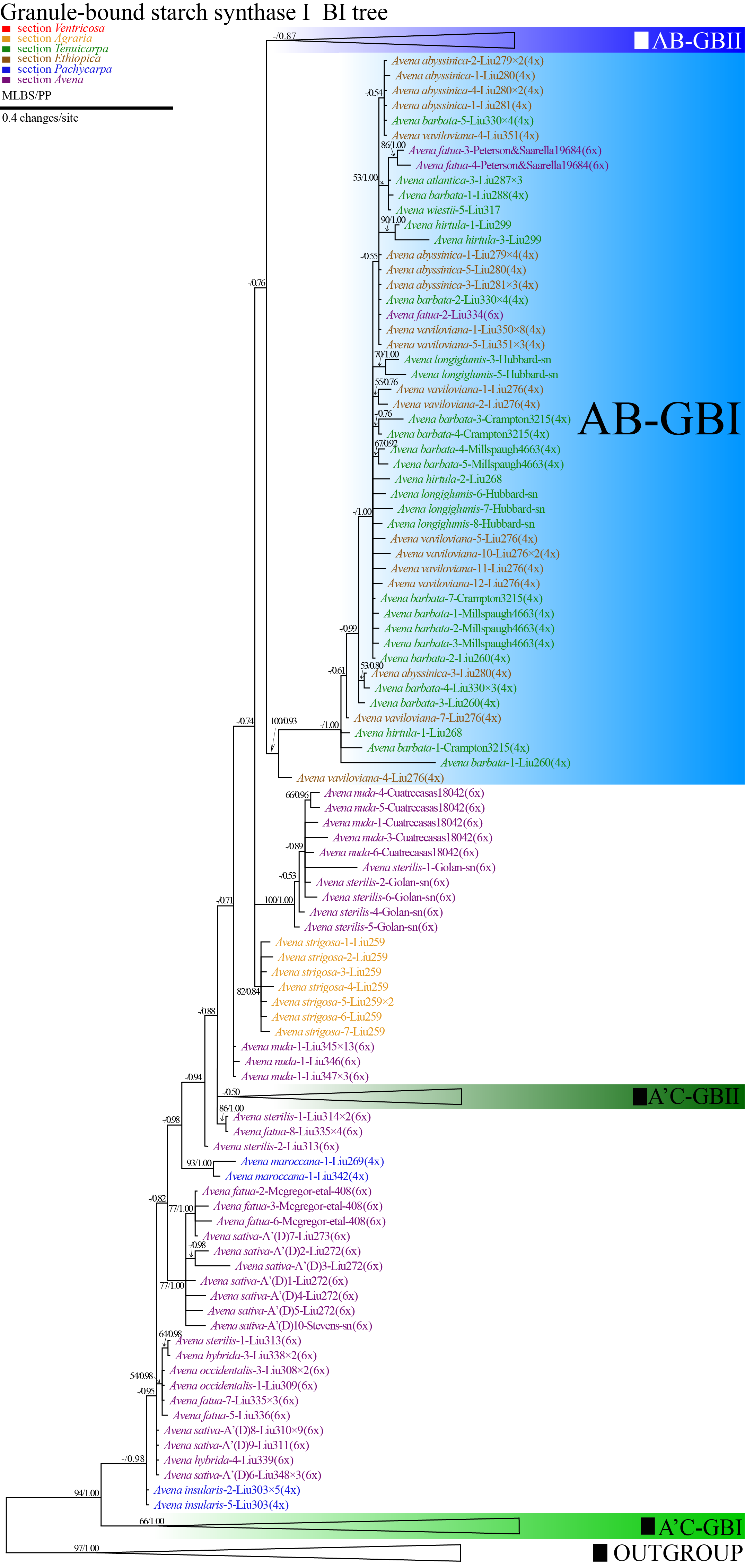

Supplement: Supplementary Figs. S1-S11 [file srep41944-s2.zip › Fig. S9AB-GBI.tif]
